# Disparity in survival benefits of pembrolizumab between Asian and non‐Asian patients with advanced cancers: A systematic review and meta‐regression analysis

**DOI:** 10.1002/cam4.6563

**Published:** 2023-09-22

**Authors:** Shang‐Hsuan Peng, Ching‐Hung Lin, I‐Chun Chen, Ying‐Chun Shen, Dwan‐Ying Chang, Tom Wei‐Wu Chen, Shu‐Min Huang, Fu‐Chang Hu, Yen‐Shen Lu

**Affiliations:** ^1^ Department of Oncology National Taiwan University Hospital Yunlin Branch Yunlin Taiwan; ^2^ Department of Medical Oncology National Taiwan University Cancer Center Taipei Taiwan; ^3^ Department of Oncology National Taiwan University Hospital Taipei Taiwan; ^4^ Graduate Institute of Clinical Medicine and School of Nursing, College of Medicine, National Taiwan University Taipei Taiwan; ^5^ Statistical Consulting Clinic International‐Harvard (I‐H) Statistical Consulting Company Taipei Taiwan

**Keywords:** advanced cancers, Asian, meta‐analysis, overall survival, pembrolizumab

## Abstract

**Background:**

Immune checkpoint inhibitors have revolutionized the treatment of malignancies. However, disproportionate enrollment among races and ethnicities places the generalizability of global trial results in doubt.

**Methods:**

In this systematic review, phase 3 randomized controlled trials investigating pembrolizumab in advanced cancers and providing subgroup analyses of Asian and non‐Asian participants were included. The primary and secondary effect measures were the mean differences (MDs) in the natural logarithms of the hazard ratios (HRs) for overall survival (OS) and progression‐free survival (PFS) between these two subgroups, respectively. We used random‐effects meta‐analysis to calculate the pooled ratios of HRs (i.e., exp(MD)) and implemented a meta‐regression analysis to identify significant covariates.

**Results:**

A total of 17 and 11 trials were included in the meta‐analyses of OS and PFS, respectively. These trials included 2732 (25.49%) Asian and 7000 (65.32%) non‐Asian participants in the OS analysis and 1438 (22.5%) Asian and 4129 (64.61%) non‐Asian participants in the PFS analysis. The pooled ratio of HRs for OS was 0.87 (95% CI: 0.76–0.99; *p* = 0.0391), favoring Asian participants, but no significant difference was found in PFS (pooled ratio of HRs: 0.93; 95% CI: 0.82–1.07; *p* = 0.2391). Both linear meta‐regression analyses revealed an open‐label design as a crucial covariate, which indicated more benefits for non‐Asian participants.

**Conclusions:**

Compared with non‐Asian patients, Asian patients with advanced cancers may derive superior OS benefits from pembrolizumab. Although the results warrant further exploration, this meta‐analysis provides insight into clinical research design.

## 1 INTRODUCTION

Recent advances in immunotherapy have revolutionized the treatment landscape for multiple cancer types, and immune checkpoint inhibitors (ICIs) are one of the most promising therapeutics. Clinical trials have repeatedly demonstrated the overall survival (OS) benefits of ICIs in patients with cancer of various types, and health authorities worldwide have granted regular approval for several ICIs, including a cytotoxic T‐lymphocyte antigen 4 (CTLA4) inhibitor (ipilimumab); programmed cell death 1 (PD‐1) inhibitors (cemiplimab, dostarlimab, nivolumab, and pembrolizumab); and programmed cell death ligand 1 (PD‐L1) inhibitors (atezolizumab, avelumab, and durvalumab). Among these, pembrolizumab is the most widely indicated and is anticipated to be the most sold drug in 2023.^1^

Patients from different racial and ethnic groups experience different epidemiology, management, and survival outcomes in various cancers.^2^ How the efficacy of ICIs varies with race and ethnicity has not been elucidated. Unique racial or ethnic features may aid in identifying potential biomarkers and advancing precision medicine. The POPLAR and OAK studies, in which patients with non‐small cell lung cancer (NSCLC) were treated with atezolizumab, reported favorable prognoses in Asian subgroups; different frequencies of specific genetic alterations were suggested to be an explanation for these results.^3^ Several molecular profiling studies of breast cancer have reported more immune‐active tumor microenvironments in Asian patients than in White patients.^4^, ^5^, ^6^ By contrast, non‐Asian patients with gastric cancers have exhibited more immune‐supportive antitumor signatures than Asian patients.^7^ However, the pharmacokinetics of pembrolizumab are similar between Asian and White patients.^8^, ^9^ The findings mentioned above shed light on the value of racial and ethnic differences in trial design for immuno‐oncology.

Racial and ethnic disparities in global trial enrollment could result in disproportionately more research on Western patients than on Eastern patients. Most trial participants are recruited from North America or Western Europe, and few participants are recruited from Asia.^10^ Most global trials have reported the results of racial and ethnic subgroup analyses, but no studies have convincingly demonstrated that the efficacy of ICIs for Asian and non‐Asian patients differs. Although some trials have indicated superior efficacy in Asian patients,^11^, ^12^, ^13^ within‐study comparisons have complicated these findings.^14^ Therefore, we performed a systematic review and meta‐analysis to examine whether the efficacy of pembrolizumab for Asian and non‐Asian patients differs significantly across various types of advanced metastatic cancers.

## 2 METHODS

This systematic review and meta‐analysis followed the Preferred Reporting Items for Systematic Reviews and Meta‐analyses (PRISMA) guidelines.^15^, ^16^

### 2.1 Literature search

Two authors, SH Peng and YS Lu, independently searched the medical literature databases of PubMed, Cochrane Library, Embase, and Web of Science for relevant articles using the terms “pembrolizumab” and “randomized controlled trial.” The details of the search strategies used for each database are provided in Table S1. Studies were included in our meta‐analysis if they (1) were phase 3 trials investigating the efficacy of pembrolizumab in a palliative setting and (2) included subgroup analysis by race or ethnicity. Phase 2 studies, trials in adjuvant or neoadjuvant settings, and irrelevant studies were excluded. We (SH Peng and YS Lu) discussed and resolved selection discrepancies with other authors until a consensus was reached. The Cochrane tool for randomized trials was used to assess the risk of bias in the following domains: the randomization process, deviations from the intended intervention, missing outcome data, outcome measurement, and selective reporting (Table S2).^17^

### 2.2 Data extraction

SH Peng and YS Lu independently extracted and verified relevant information from the published manuscripts and supplementary appendices of 26 selected studies to obtain the following metadata: trial name, first author, publication year, study design (open‐label or double‐blind, placebo‐controlled), cancer type, setting (first‐line treatment or beyond), experimental treatment strategy (monotherapy, add‐on, or combination), criteria for PD‐L1 expression, primary endpoints, patient numbers, and demographic characteristics (e.g., median age, sex distribution, and the percentage of Eastern Cooperative Oncology Group performance status [ECOG PS] scores of 0). The selected cutoffs for PD‐L1 expression agreed with the primary analysis by statistical testing hierarchy, available demographic characteristics, or label indications (Table S3).

The experimental treatment strategies were categorized as (1) monotherapy versus standard of care (SOC), (2) monotherapy versus best supportive care (BSC), (3) add‐on to chemotherapy (ChT) versus SOC, (4) add‐on to multikinase inhibitor (MKI) versus SOC; and (5) combined therapy with MKI versus standard ChT. Cancer types were classified into nine groups: (1) NSCLC, (2) hepatocellular carcinoma (HCC), (3) squamous cell carcinoma of the aerodigestive tract, (4) esophageal and gastric adenocarcinoma, (5) breast cancer, (6) renal cell carcinoma (RCC), (7) melanoma, (8) urothelial carcinoma (UC), and (9) miscellaneous cancers. We also collected the estimated hazard ratios (HRs) with 95% CIs for OS and progression‐free survival (PFS) for the total populations, Asian subgroups, and non‐Asian subgroups.

### 2.3 Statistical analysis

We used the metafor package (version 3.8‐1) in R 4.2.1 (R Foundation for Statistical Computing) to perform meta‐analyses and meta‐regression analyses.^18^ A two‐sided *p* ≤ 0.05 was considered statistically significant. The primary and secondary outcomes were OS and PFS, respectively. The mean difference (MD) in the natural logarithms of the HRs for OS or PFS between the Asian and non‐Asian subgroups was determined as the effect measure to compare the efficacy of pembrolizumab in the Asian and non‐Asian subgroups, as the following equation:

$$\mathrm{MD}=\log\left({\mathrm{HR}}_{\text{Asian}}\right)-\log\left({\mathrm{HR}}_{\text{Non}-\text{Asian}}\right)=\log\left[\frac{{\mathrm{HR}}_{\text{Asian}}}{{\mathrm{HR}}_{\text{Non}-\text{Asian}}}\right]$$

where log is the natural logarithm; MD values <0 and >0 imply that pembrolizumab treatment benefits Asian and non‐Asian patients more, respectively.

In forest plots, the estimated pooled ratios of HRs (i.e., exp(MD)) for OS and PFS for Asian to non‐Asian patients were obtained through random‐effects meta‐analysis with Knapp–Hartung adjustment. The heterogeneity among the selected studies was evaluated using the chi‐square *Q* test and the *I* ^2^ statistic, where a *p* < 0.15 in the *Q* test or *I* ^2^ of >50% indicated substantial heterogeneity.^19^

If the statistical tests for heterogeneity and the *I* ^2^ or substantive knowledge suggested substantial heterogeneity among the analyzed studies, a fixed‐effects linear meta‐regression analysis of MDs was fitted to the metadata through the weighted least‐squares method to identify the relevant covariate that might account for the observed heterogeneity. If the residual heterogeneity remained statistically significant, a mixed‐effects linear meta‐regression analysis of the MDs was performed with added random effects to account for unknown sources of heterogeneity.

Simple and multiple generalized additive models (GAMs) of the MDs were constructed and used to draw GAM plots for detecting the nonlinear effects of continuous covariates and identifying the appropriate cutoffs for continuous covariates, if necessary, during the stepwise variable selection procedure. We used the vgam() function with the default values of the smoothing parameters (e.g., s(age, df = 4, spar = 0) for *cubic smoothing splines*) in the VGAM package in R to fit the GAMs for continuous responses and then used the plotvgam() function of the same package to draw GAM plots to visualize the linear or nonlinear effects of continuous covariates.^20^

Variable selection, goodness‐of‐fit assessment, and regression diagnostics were used in the meta‐regression analysis to ensure the quality of the results. Specifically, with the aid of the likelihood ratio test, a stepwise variable selection procedure (including iterations between the *forward* and *backward* steps) was implemented to obtain the candidate variables for the final linear meta‐regression model. The coefficient of determination, *R* ^2^, for the final model is the square of the Pearson's correlation coefficient between the observed and predicted response values. Finally, regression diagnostics were performed, including multicollinearity, residual analysis, influential studies identification, and publication bias assessments.

## 3 RESULTS

### 3.1 Literature search results

**FIGURE 1:**
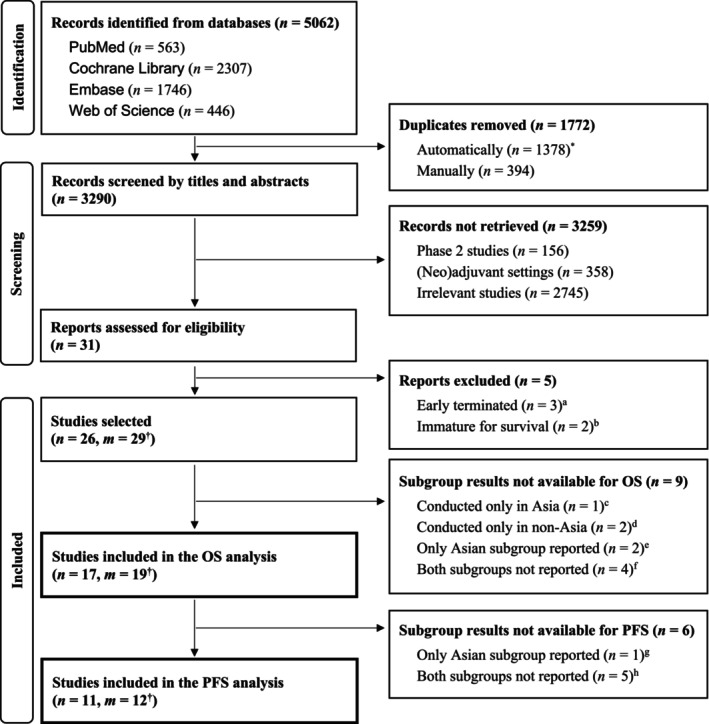
PRISMA flow diagram of study selection. OS, overall survival; PFS, progression‐free survival. *Duplicates were removed automatically by the EndNote^TM^ software (version 20.3, 2022). ^†^KEYNOTE (KN)‐048, ‐062, and ‐361 had three arms. ^a^KN‐063 (gastric cancer), ‐183 (multiple myeloma), and ‐185 (multiple myeloma). ^b^KN‐204 (Hodgkin's lymphoma) and ‐811 (gastric cancer). ^c^KN‐394 (hepatocellular carcinoma). ^d^KN‐006 (melanoma) and ETOP 9–15 PROMISE‐meso (pleural mesothelioma). ^e^KN‐045 (urothelial carcinoma) and ‐189 (non‐small cell lung cancer, adenocarcinoma). ^f^KN‐040 (head and neck squamous cell carcinoma), ‐119 (breast cancer), ‐361 (urothelial carcinoma), and ‐826 (cervical cancer). ^g^KN‐062 pembrolizumab monotherapy arm (gastric cancer). ^h^KN‐010 (non‐small cell lung cancer), ‐042 (non‐small cell lung cancer), ‐061 (gastric cancer), ‐122 (nasopharyngeal cancer), ‐181 (esophageal cancer).

The flow diagram in Figure 1 depicts the study selection process. A total of 5062 publications were initially identified; 3290 remained after duplicates were removed, and their titles and abstracts were screened. The main texts from 31 randomized controlled trials (RCTs) were thoroughly reviewed for eligibility, of which five were excluded because of early termination (*n* = 3)^21^, ^22^, ^23^ or immature survival data (*n* = 2).^24^, ^25^ Among the 26 remaining eligible studies (i.e., *n* = 26), three contained 3 trial arms (i.e., *m* = 29).^13^, ^26^, ^27^ The results of racial or ethnic subgroup analyses for OS were unavailable for nine trials,^27^, ^28^, ^29^, ^30^, ^31^, ^32^, ^33^, ^34^, ^35^, ^36^, ^37^, ^38^, ^39^, ^40^, ^41^, ^42^ and those for PFS were unavailable for six trials.^11^, ^13^, ^43^, ^44^, ^45^, ^46^, ^47^, ^48^, ^49^ The remaining trials were included in the meta‐analysis (*n* = 17, *m* = 19 for OS^11^, ^12^, ^13^, ^26^, ^44^, ^45^, ^46^, ^47^, ^48^, ^49^, ^50^, ^51^, ^52^, ^53^, ^54^, ^55^, ^56^, ^57^, ^58^, ^59^, ^60^, ^61^, ^62^, ^63^, ^64^, ^65^ and *n* = 11, *m* = 12 for PFS^12^, ^26^, ^50^, ^51^, ^52^, ^53^, ^54^, ^55^, ^56^, ^57^, ^58^, ^59^, ^60^, ^61^, ^62^, ^63^, ^64^, ^65^).

### 3.2 Characteristics of selected studies

Table 1 provides an overview of the 26 selected studies, including the trial and demographic characteristics. Every trial recruited patients within the last decade, and most studies were published in the past 2 years. The studies investigated several cancer types, the most common being NSCLC (five trials),^37^, ^45^, ^46^, ^51^, ^52^ followed by HCC,^28^, ^53^ head and neck squamous cell carcinoma (HNSCC),^26^, ^39^ esophageal cancer,^11^, ^12^ gastric cancer,^13^, ^48^ advanced breast cancer,^40^, ^56^ clear cell RCC,^58^, ^60^ and UC^27^, ^34^ (two trials each). Other cancer types were melanoma,^31^ nasopharyngeal cancer,^49^ microsatellite instability‐high colorectal cancer,^63^ SCLC,^64^ endometrial cancer,^65^ cervical cancer,^42^ and pleural mesothelioma^32^ (one trial each). Nine trials (34.62%) used a double‐blind, placebo‐controlled design.^12^, ^13^, ^28^, ^37^, ^42^, ^52^, ^53^, ^56^, ^64^ In total, 14 (53.85%) were in only first‐line settings^12^, ^13^, ^26^, ^27^, ^37^, ^42^, ^46^, ^51^, ^52^, ^56^, ^58^, ^60^, ^63^, ^64^ and one (3.85%) was in first‐ and second‐line settings.^31^ Of 29 paired arms, 15 (51.72%) compared monotherapy and SOC,^11^, ^13^, ^26^, ^27^, ^31^, ^32^, ^34^, ^39^, ^40^, ^45^, ^46^, ^48^, ^49^, ^51^, ^63^ two (6.90%) compared monotherapy and BSC,^28^, ^53^ nine (31.03%) compared an add‐on to ChT and SOC,^12^, ^13^, ^26^, ^27^, ^37^, ^42^, ^52^, ^56^, ^64^ two (6.90%) compared an add‐on to MKI and SOC,^58^, ^60^ and one (3.45%) compared combination pembrolizumab‐MKI and ChT.^65^ Regarding the selected cutoffs of PD‐L1 expression (Table S3), most studies were all‐comer trials (*n* = 18, 69.23%),^11^, ^12^, ^26^, ^27^, ^28^, ^31^, ^32^, ^34^, ^37^, ^39^, ^49^, ^52^, ^53^, ^58^, ^60^, ^63^, ^64^, ^65^ regardless of PD‐L1 status; the other trials were a tumor proportion score (TPS) ≥1 (*n* = 2),^45^, ^46^ a TPS ≥50 (*n* = 1),^51^ a combined positive score (CPS) ≥1 (*n* = 3),^13^, ^42^, ^48^ or a CPS ≥10 (*n* = 2).^40^, ^56^ OS was used as a secondary endpoint in three trials^32^, ^51^, ^60^ and as a primary endpoint (either single or multiple) in the remaining trials. The overall median age in trial arms ranged from 50 to 71 years, with the differences between paired arms being −3 to 3 years. The percentage of men ranged from 0% to 87.1%, with differences from −7.3% to 9.0%. Finally, the percentage of ECOG PS scores of 0 ranged from 19.7% to 83.1%, with differences from −5.8% to 9.4%.

**TABLE 1:** Trial and demographic characteristics of selected studies.

Cancer group	First author (year)	Trial name	Cancer type	Setting	Experimental treatment	Control treatment	PD‐L1 expression	Primary endpoints	Maximal median follow‐up in months (range)
1	Herbst (2021)^45^	KN‐010	NSCLC	2L	Monotherapy	ChT^1^	TPS1	PFS/OS	67.4 (60.0–77.9)
	Reck (2019)^51^	KN‐024	NSCLC	1L	Monotherapy	ChT^1^	TPS50	PFS	25.2 (20.4–33.7)
	Mok (2019)^46^	KN‐042	NSCLC	1L	Monotherapy	ChT^1^	TPS1	OS	12.8 (6.00–20.0)
	Rodríguez‐Abreu (2021)^37^	KN‐189^a^	NSCLC‐ADC	1L	Add‐on to ChT	ChT^3^	All comers	PFS/OS	31.0 (26.5–38.8)
	Paz‐Ares (2018)^52^	KN‐407^a^	NSCLC‐SQ	1L	Add‐on to ChT	ChT^3^	All comers	PFS/OS	7.8 (0.10–19.1)
2	Finn (2020)^53^	KN‐240^a^	HCC	2L	Monotherapy	BSC^2^	All comers	PFS/OS	13.8 (0.90–30.4)
	Qin (2022)^28^	KN‐394^a^	HCC	2L	Monotherapy	BSC^2^	All comers	OS	33.8 (18.7–49.0)
3	Cohen (2019)^39^	KN‐040	HNSCC	2/3L	Monotherapy	ChT^1^	All comers	OS	7.5 (3.40–13.3)
	Burtness (2019)^26^	KN‐048‐P	HNSCC	1L	Monotherapy	ChT + cetuximab^1^	All comers	PFS/OS	13.0 (6.40–26.6)
		KN‐048‐C			Add‐on to ChT	ChT + cetuximab^3^			
3 (2/3)^b^ 4 (1/3)^b^	Kojima (2020)^11^	KN‐181	EC	2L	Monotherapy	ChT^1^	All comers^c^	OS	7.1 (0.50–31.3)
	Sun (2021)^12^	KN‐590^a^	EC	1L	Add‐on to ChT	ChT^3^	All comers	PFS/OS	22.6 (19.6–27.1)
4	Fuchs (2022)^48^	KN‐061	GC	2L	Monotherapy	ChT^1^	CPS1	PFS/OS	32.5 (NR)
	Shitara (2020)^13^	KN‐062‐P	GC	1L	Monotherapy	ChT^1^	CPS1	PFS/OS	29.4 (22.0–41.3)
		KN‐062‐C^a^			Add‐on to ChT	ChT^3^			
5	Winer (2021)^40^	KN‐119	ABC	2/3L	Monotherapy	ChT^1^	CPS10	OS	31.5 (27.8–34.6)
	Cortés (2021)^56^	KN‐355^a^	ABC	1L	Add‐on to ChT	ChT^3^	CPS10	PFS/OS	44.4 (NR)
6	Powles (2020)^58^	KN‐426	ccRCC	1L	Add‐on to MKI	MKI^4^	All comers	PFS/OS	30.6 (23.4–38.4)
	Motzer (2021)^60^	KN‐581	ccRCC	1L	Add‐on to MKI	MKI^4^	All comers	PFS	26.6 (NR)
7	Robert (2019)^31^	KN‐006	Melanoma	1/2L	Monotherapy	Ipilimumab^1^	All comers	PFS/OS	57.7 (56.7–59.2)
8	Fradet (2019)^34^	KN‐045	UC	2L	Monotherapy	ChT^1^	All comers	PFS/OS	27.7 (NR)
	Powles (2021)^27^	KN‐361‐P	UC	1L	Monotherapy	ChT^1^	All comers	PFS/OS	31.7 (27.7–36.0)
		KN‐361‐C			Add‐on to ChT	ChT^3^			
9	Chan (2021)^49^	KN‐122	NPC	2/3L	Monotherapy	ChT^1^	All comers	OS	45.1 (30.2–54.8)
	Diaz Jr (2022)^63^	KN‐177	MSI‐H CRC	1L	Monotherapy	ChT + biologics^1^	All comers	PFS/OS	44.5 (39.7–49.8)
	Rudin (2020)^64^	KN‐604^a^	SCLC	1L	Add‐on to ChT	ChT^3^	All comers	PFS/OS	21.6 (16.1–30.6)
	Makker (2022)^65^	KN‐775	EMC	2/3L	Combination	ChT^5^	All comers	PFS/OS	12.2 (NR)
	Colombo (2021)^42^	KN‐826^a^	CC	1L	Add‐on to ChT	ChT ± bevacizumab^3^	CPS1	PFS/OS	22.0 (15.1–29.4)
	Popat (2020)^32^	ETOP 9–15	PM	2L	Monotherapy	ChT^1^	All comers	PFS	17.5 (14.8–19.7)

Median age (range)			Men (%)			ECOG PS scores of 0 (%)		
Experimental	Control	Difference	Experimental	Control	Difference	Experimental	Control	Difference
63 (56–69)	62 (56–69)	1.0	61.6	60.9	0.7	33.5	33.8	−0.3
64.5 (33–90)	66 (38–85)	−1.5	59.7	62.9	−3.2	35.1	35.1	0.0
63 (57–69)	63 (57–69)	0.0	70.6	71.0	−0.3	31.1	30.1	0.9
65 (34–84)	63.5 (34–84)	1.5	62.0	52.9	9.0	45.4	38.8	6.5
65 (29–87)	65 (36–88)	0.0	79.1	83.6	−4.5	26.3	32.0	−5.8
67 (18–91)	65 (23–89)	2.0	81.3	83.0	−1.7	58.3	52.6	5.7
54 (22–82)	54 (22–78)	0.0	85.7	82.4	3.3	41.3	39.2	2.1
60 (55–66)	60 (54–66)	0.0	83.8	82.7	1.1	28.7	27.0	1.7
62 (56–68)	61 (54.5–68)	1.0	83.1	87.0	−3.9	39.2	39.0	0.2
61 (55–68)	61 (55–68)	0.0	79.7	87.1	−7.3	39.1	38.8	0.3
63 (23–84)	62 (24–84)	1.0	86.9	86.3	0.6	40.1	36.9	3.2
64 (28–94)	62 (27–89)	2.0	82.0	84.8	−2.8	39.9	39.9	0.1
64 (33–87)	61 (24–86)	3.0	74.5	70.4	4.1	44.9	46.2	−1.3
61 (20–83)	62.5 (23–87)	−1.5	70.3	71.6	−1.3	51.2	46.0	5.2
62 (22–83)	62.5 (23–87)	−0.5	75.9	71.6	4.3	46.3	46.0	0.3
50 (28–85)	50.5 (31–79)	−0.5	0.0	0.0	0.0	60.4	51.0	9.4
52 (44–62)	55 (43–63)	−3.0	0.0	0.0	0.0	60.9	60.2	0.7
62 (30–89)	61 (26–90)	1.0	71.3	74.6	−3.3	80.1	79.5	0.6
64 (34–88)	61 (29–82)	3.0	71.8	77.0	−5.2	83.1	82.4	0.7
62 (18–89)	62 (18–88)	0.0	60.3	58.3	2.0	69.1	67.6	1.4
67 (29–88)	65 (26–84)	2.0	74.1	74.3	−0.2	44.4	39.0	5.5
68 (61–74)	69 (61–75)	−1.0	74.3	74.4	−0.2	43.6	47.7	−4.1
69 (62–75)	69 (61–75)	0.0	77.5	74.4	3.1	42.7	47.7	−5.0
51 (21–76)	53 (23–78)	−2.0	83.8	81.9	1.9	30.8	33.6	−2.9
63 (52–73)	63 (48–72)	0.0	46.4	53.2	−6.8	49.0	54.5	−5.5
64 (24–81)	65 (37–83)	−1.0	66.7	63.1	3.6	26.3	24.9	1.4
64 (30–82)	65 (35–86)	−1.0	0.00	0.00	0.0	59.9	57.9	1.9
51 (25–82)	51 (22–78)	0.0	0.00	0.00	0.0	58.6	53.8	4.8
69 (52–83)	71 (53–83)	−2.0	79.5	84.5	−5.1	28.8	19.7	9.0

*Note*: ^1–5^ Treatment strategies were categorized as (1) monotherapy versus standard of care (SOC), (2) monotherapy versus BSC, (3) add‐on to ChT versus SOC, (4) add‐on to MKI versus SOC, and (5) combined therapy with MKI versus standard ChT.

Abbreviations: 1/2/3L, first‐/second‐/third‐line; ABC, advanced breast cancer; ADC, adenocarcinoma; BSC, best supportive care; C, combination arm; CC, cervical cancer; ccRCC, clear cell renal cell carcinoma; ChT, chemotherapy; CPS, combined positive score; CRC, colorectal cancer; EC, esophageal cancer; ECOG PS, Eastern Cooperative Oncology Group performance status; EMC, endometrial cancer; ETOP, European Thoracic Oncology Platform; GC, gastric cancer; HCC, hepatocellular carcinoma; HNSCC, head and neck squamous cell carcinoma; KN, KEYNOTE; MKI, multikinase inhibitor; MSI‐H, microsatellite instability‐high; NPC, nasopharyngeal cancer; NR, not reported; NSCLC, non‐small cell lung cancer; OS, overall survival; P, pembrolizumab monotherapy arm; PD‐L1, programmed death ligand‐1; PFS, progression‐free survival; PM, pleural mesothelioma; SQ, squamous cell carcinoma; TPS, tumor proportion score; UC, urothelial carcinoma.

^a^ Double‐blind, placebo‐controlled design (*n* = 9); KN‐062 was a partially blinded trial.

^b^ The percentage of patients with squamous cell carcinoma was 64.9% in KN‐181 and 73.2% in KN‐590. The remaining approximately one‐third of patients had ADC.

^c^ Although the cutoff of PD‐L1 expression was CPS10 according to the statistical testing hierarchy in KN‐181, the demographic characteristics of this subset of patients were unavailable. Thus, we used the intention‐to‐treat population.

### 3.3 Asian and non‐Asian subgroups

A total of 15,553 patients were included in the selected studies (*n* = 26). Of these, 8814 received pembrolizumab, and 6739 received control treatments. Among all patients, 23.01% (3579) were Asian (Table 2). Subgroup analyses based on race or ethnicity were available for OS for 17 trials and PFS for 11 trials and comprised 10,717 and 6391 patients, respectively. In the OS analysis, the Asian and non‐Asian subgroups had 2732 (25.49%) and 7000 (65.32%) patients, respectively. In the PFS analysis, the Asian and non‐Asian subgroups had 1438 (22.50%) and 4129 (64.61%) patients, respectively.

**TABLE 2:** Patient numbers in total populations, Asian subgroups, and non‐Asian subgroups.

First author (year)	Trial name	Total population			Asian subgroup				Non‐Asian subgroup^a^			
		Exp	Ctrl	Total	Exp	Ctrl	Total	(%)^b^	Exp	Ctrl	Total	(%)^b^
Herbst (2021)^45^	KN‐010	690	343	1033	128	62	190	18.39	562	281	843	81.61
Reck (2019)^51^	KN‐024	154	151	305	21^c^	19^c^	40	13.11	133	132	265	86.89
Mok (2019)^46^	KN‐042	637	637	1274	185	185	370	29.04	452	452	904	70.96
*Horinouchi (2021)* ^38^	KN‐189	410	206	616	25^c^	15^c^	40	6.49	N/A	N/A	N/A	N/A
Paz‐Ares (2018)^52^	KN‐407	278	281	559	54	52	106	18.96	224	229	453	81.04
Finn (2020)^53^	KN‐240	278	135	413	67^c^	31^c^	98	23.73	211	104	315	76.27
Qin (2022)^28^	KN‐394	300	153	453	300	153	453	100.00	N/A	N/A	0	0.00
Cohen (2019)^39^	KN‐040	247	248	495	N/A	N/A	22	4.44	N/A	N/A	N/A	N/A
*Ngamphaiboon (2019)* ^54^	KN‐048‐P	301	300	601	56	53	109	18.14	245	247	492	81.86
	KN‐048‐C	281	278^d^	559	57	49^d^	106	18.96	224	229^d^	453	81.04
Kojima (2020)^11^	KN‐181	314	314	628	121	122	243	38.69	193	192	385	61.31
Sun (2021)^12^	KN‐590	373	376	749	196^c^	197^c^	393	52.47	177	179	356	47.53
Fuchs (2022)^48^	KN‐061	196	199	395	52	52	104	26.33	131	132	263	66.58
Satake (2020)^43^	KN‐062‐P	256	250	506	62	61	123	24.31	148	147	295	58.30
Shitara (2020)^13^	KN‐062‐C	257		507	64		125	24.65	148		295	58.19
*Im (2020)* ^41^	KN‐119	96	98	194	22^e^	24^e^	46^e^	23.71	N/A	N/A	N/A	N/A
Cortés (2021)^56^	KN‐355	220	103	323	38	18	56	17.34	N/A^e^	N/A^e^	212^e^	65.63
*Kondoh (2020)* ^59^	KN‐426	432	429	861	62	68	130	15.10	161	156	317	36.82
*Rha (2022)* ^61^	KN‐581	355	357	712	75	65	140	19.66	198	199	397	55.76
Robert (2019)^31^	KN‐006	556	278	834	N/A	N/A	0	0.00	N/A	N/A	N/A	N/A
*Nishiyama (2020)* ^35^	KN‐045^f^	270	272	542	30^c^	22^c^	113^f^	20.85	N/A	N/A	N/A	N/A
Powles (2021)^27^	KN‐361‐P	307	352	659	N/A	N/A	173	17.13	N/A	N/A	N/A	N/A
	KN‐361‐C	351		703	N/A				N/A		N/A	
Chan (2021)^49^	KN‐122	117	116	233	95	105	200	85.84	22	11	33	14.16
Diaz Jr (2022)^63^	KN‐177	153	154	307	22	26	48	15.64	109	113	222	72.31
Rudin (2020)^64^	KN‐604	228	225	453	52	32	84	18.54	176	193	369	81.46
Makker (2022)^65^	KN‐775	411	416	827	85^c^	92^c^	177	21.40	261	246	507	61.31
Colombo (2021)^42^	KN‐826	273	275	548	N/A	N/A	N/A	N/A	N/A	N/A	N/A	N/A
Popat (2020)^32^	ETOP 9–15	73	71	144	N/A	N/A	0	0.00	N/A	N/A	N/A	N/A
Selected studies (*n* = 26, *m* = 29)	Total	8814	6739	15,553	N/A	N/A	**3579**	23.01	N/A	N/A	**N/A**	N/A
Meta‐analysis												
OS (*n* = 17, *m* = 19)	Total	5931	4786	10,717	1492	1240	**2732**	25.49	N/A	N/A	**7000**	65.32
PFS (*n* = 11, *m* = 12)	Total	3464	2927	6391	785	653	**1438**	22.50	N/A	N/A	**4129**	64.61

*Note*: Selected studies with fully reported hazard ratios (HRs) for **OS** in both subgroups are denoted by **bold** text in the Trial name column (*n* = 17, *m* = 19), among which those with HRs for **PFS** in both subgroups are denoted by underlining (*n* = 11, *m* = 12). Asian substudies are denoted by *italic* text in the First author (year) column.

Abbreviations: C, combination arm; Ctrl, control arm; ETOP, European Thoracic Oncology Platform; Exp, experimental arm; KN, KEYNOTE; N/A, not available; OS, overall survival; P, pembrolizumab monotherapy arm; PFS, progression‐free survival.

^a^ The composition of each non‐Asian subgroup is specified in Table S4.

^b^ Total patient numbers of Asian subsets divided by entire trial populations, unless otherwise specified.

^c^ Except for KN‐775 stratified by race (155 patients from East Asia and 22 patients from non‐Asian countries), all selected studies were stratified by geographic region. The reported Asian subgroups contained only Japanese patients (KN‐024, ‐045 and ‐189), East Asian patients without Japanese patients (KN‐240), and East Asian patients without Thai or Malaysian patients (KN‐590). Otherwise, the Asian subgroup was defined as those patients enrolled from countries in East Asia (Table S4).

^d^ In KN‐048, the enrollment of the combination arm had been temporarily postponed, and the 22 patients allocated to the control arm during this period were excluded.

^e^ In the Asian subgroup of KN‐119, patient numbers were reported, but the HR for OS was not available. In the non‐Asian subgroup of KN‐355, the HR for OS was available, but patient numbers were not.

^f^ In KN‐045, 113 patients were enrolled from East Asia.

Table S4 lists the countries participating in each trial. The top three Asian countries participating in global trials were Japan, South Korea, and Taiwan. The non‐Asian countries for global trials were in North America (Canada and the United States), Europe (mainly France, Germany, Spain, and the United Kingdom), Oceania (Australia and New Zealand), Africa (South Africa), the Middle East (Israel and Turkey), and Latin America (mainly Argentina and Chile).

### 3.4 Primary analysis (OS)

**FIGURE 2:**
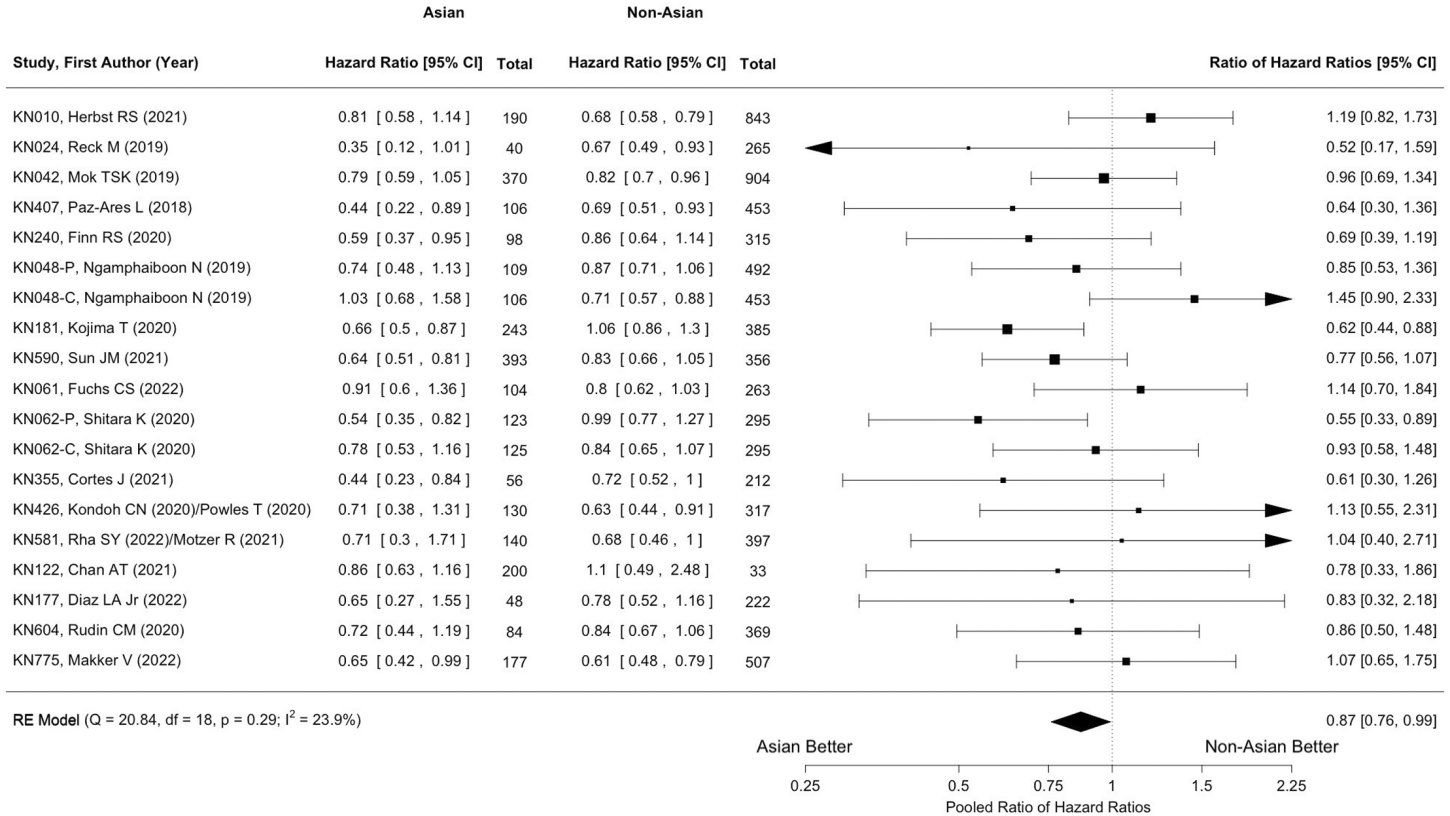
Random‐effects meta‐analysis of overall survival differences with Knapp‐Hartung adjustment (*m* = 19). The pooled ratio of HRs for OS between the Asian and non‐Asian subgroups (0.87; 95% CI, 0.76–0.99; *p* = 0.0391; chi‐square *Q* test *χ* ^2^ = 20.84; *p* = 0.29; *I* ^2^ = 23.9%). Each paired arm corresponds to a square centered at the point estimate of the effect measure (i.e., the ratio of the HRs of the Asian to non‐Asian subgroup), with the size being proportional to the study weight. The horizontal line extending from both sides of the square represents the 95% CI. “RE” denotes “random‐effects.”

The random‐effects meta‐analysis with Knapp–Hartung adjustment (*m* = 29) revealed that pembrolizumab had a statistically significant OS benefit in the total populations (HR: 0.76; 95% CI: 0.73–0.80; *p* < 0.001; Figure S1A); heterogeneity was statistically significant (chi‐square *Q* test *χ* ^2^ = 44.22; *p* = 0.03; *I* ^2^ = 36.6%). The pooled ratio of HRs for OS between the Asian and non‐Asian subgroups (*m* = 19) also revealed a statistically significant difference (HR: 0.87; 95% CI: 0.76–0.99; *p* = 0.0391; chi‐square *Q* test *χ* ^2^ = 20.84, *p* = 0.29; *I* ^2^ = 23.9%; Figure 2).

Table 3A summarizes the results of the linear fixed‐effects meta‐regression analysis of the MDs in the natural logarithms of the HRs for OS between the Asian and non‐Asian subgroups. After adjustment for the effects of other covariates, the mean value of the MDs was −0.7424, favoring the Asian subgroups (*p* < 0.001; estimated ratio of HRs: 0.4760; 95% CI: 0.3261–0.6948). This value was 0.4897 higher in the trials using add‐on pembrolizumab (to ChT or MKI) or combination pembrolizumab‐MKI with an open‐label design (*p* = 0.0069; estimated ratio of HRs: 1.6318; 95% CI: 1.1442–2.3273), 0.4257 higher if the percentage of ECOG PS scores of 0 was ≤50.28% in the experimental arm (*p* = 0.0085; estimated ratio of HRs: 1.5307; 95% CI: 1.1148–2.1018), and 0.2787 higher if the percentage of men was ≤81.92% in the experimental arm (*p* = 0.0411; estimated ratio of HRs: 1.3214; 95% CI: 1.0113–1.7265).

**TABLE 3:** Linear fixed‐effects meta‐regression analysis of mean differences in natural logarithms of hazard ratios for overall survival (A) and progression‐free survival (B) between Asian and non‐Asian subgroups.

Covariates	Regression coefficient estimate	Standard error	*Z* value	*p* value	Estimated ratio of hazard ratios (95% CI)
(A) MD for overall survival (*m* = 19)^a^					
Intercept	−0.7424	0.1930	−3.8468	0.0001	0.4760 (0.3261–0.6948)
Add‐on (to ChT or MKI) or combination with open‐label design	0.4897	0.1811	2.7036	0.0069	1.6318 (1.1442–2.3273)
ECOG PS scores of 0 ≤50.28% (experimental arm)	0.4257	0.1618	2.6316	0.0085	1.5307 (1.1148–2.1018)
Men ≤81.92% (experimental arm)	0.2787	0.1365	2.0422	0.0411	1.3214 (1.0113–1.7265)
(B) MD for progression‐free survival (*m* = 12)^b^					
Intercept	−0.2247	0.1095	−2.0518	0.0402	0.7988 (0.6444–0.9900)
Open‐label design	0.2948	0.1501	1.9646	0.0495	1.3429 (1.0007–1.8020)

*Note*: These meta‐regression analyses were performed using the escalc() and rma() functions in the metafor package (version 3.8‐1) of R‐4.2.1.

Abbreviations: ChT, chemotherapy; CI, confidence interval; ECOG PS, Eastern Cooperative Oncology Group performance status; MD, mean difference; MKI, multikinase inhibitor.

^a^ The test for residual heterogeneity showed a χ^2^ statistic (df = 15) = 6.26, *p* = 0.98 and *I* ^2^ ≈ 0%. Thus, the linear fixed‐effects meta‐regression model was reported (*R* ^2^ = 0.5171).

^b^ The test for residual heterogeneity showed a χ^2^ statistic (df = 10) = 3.51, *p* = 0.97 and *I* ^2^ ≈ 0%. Thus, the linear fixed‐effects meta‐regression model was reported (*R* ^2^ = 0.3577).

The normal *Q*–*Q* plot of the regression residuals indicated that the assumption of normality was not violated (Figure S2A). A funnel plot and Egger's test for publication bias did not demonstrate significant asymmetry (*p* = 0.3686; Figure S3A).

### 3.5 Secondary analysis (PFS)

**FIGURE 3:**
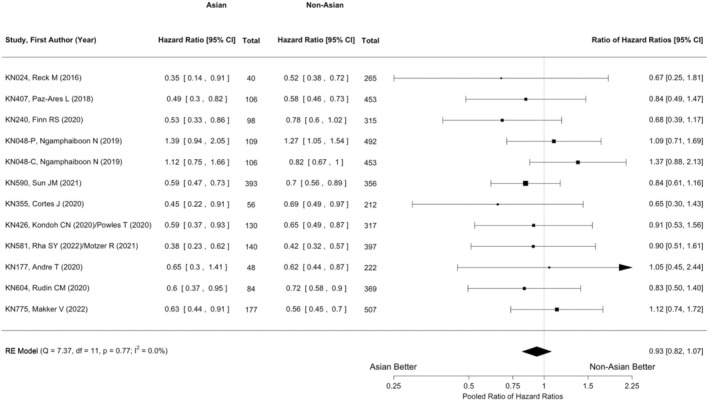
Random‐effects meta‐analysis of progression‐free survival differences with Knapp–Hartung adjustment (*m* = 12). The pooled ratio of HRs for PFS between the Asian and non‐Asian subgroups (0.93; 95% CI: 0.82–1.07; *p* = 0.2391; chi‐square *Q* test χ^2^ = 7.37; *p* = 0.77; *I* ^2^ ≈ 0%). Each paired arm corresponds to a square centered at the point estimate of the effect measure (i.e., the ratio of the HRs of the Asian to non‐Asian subgroup), with the size being proportional to the study weight. The horizontal line extending from both sides of the square represents the 95% CI. “RE” denotes “random‐effects.”

The random‐effects meta‐analysis with Knapp–Hartung adjustment (*m* = 29) revealed that the efficacy of pembrolizumab in PFS was statistically significant in the total populations (HR: 0.81; 95% CI: 0.71–0.93; *p* = 0.0039; Figure S1B). Statistically significant heterogeneity was also observed (chi‐square *Q* test χ^2^ = 329.96, *p* < 0.001; *I* ^2^ = 92.0%). The pooled ratio of HRs for PFS between the Asian and non‐Asian subgroups (*m* = 12) did not reach statistical significance (HR: 0.93; 95% CI: 0.82–1.07; *p* = 0.2391; chi‐square *Q* test χ^2^ = 7.37, *p* = 0.77; *I* ^2^ ≈ 0%; Figure 3).

Table 3B presents the results of the linear fixed‐effects meta‐regression analysis of the MDs in the natural logarithms of the HRs for PFS between the Asian and non‐Asian subgroups. After adjustment for the effects of other covariates, the mean value of the MDs was −0.2247 (*p* = 0.0402; estimated ratio of HRs: 0.7988; 95% CI: 0.6444–0.9900). This value was 0.2948 higher in open‐label trials (*p* = 0.0495; estimated ratio of HRs: 1.3429; 95% CI: 1.0007–1.8020).

The normal *Q*‐*Q* plot of the regression residuals indicated that the assumption of normality was not violated (Figure S2B). A funnel plot and Egger's test did not suggest significant publication bias (*p* = 0.2217; Figure S3B).

## 4 DISCUSSION

In this systematic review, approximately 25% of the patients in the formal analyses of OS and PFS were Asian (25.49% and 22.50%, respectively). Similar to a previous report,^66^ our meta‐analysis results suggest that Asian patients may derive a greater OS benefit from pembrolizumab than non‐Asian patients (pooled ratio of HRs: 0.87; 95% CI: 0.76–0.99; *p* = 0.0391). However, the efficacy in PFS did not significantly differ between the Asian and non‐Asian subgroups (pooled ratio of HRs: 0.93; 95% CI: 0.82–1.07; *p* = 0.2391). Generally, OS is a relatively solid endpoint in oncology trials compared to PFS. Consistent with our analysis results (Figure S1A,B), the magnitude of the effects in the PD‐(L)1 inhibitors was greater for OS than for PFS in a meta‐analysis report.^67^ The traditional assessment of PFS by the response evaluation criteria in solid tumors (version 1.1) inadequately reflects the advantage of PD‐(L)1 inhibitors, particularly in the context of atypical response patterns (e.g., pseudoprogression). Accordingly, OS is suggested to be the gold standard endpoint in PD‐(L)1 inhibitor trials.^67^ When exploring the heterogeneity accounting for the observed differences, both meta‐regression analyses identified an open‐label design as a significant contributing factor, with the regression coefficient estimates indicating more benefits for non‐Asian patients (estimated ratio of HRs for OS and PFS, 1.6318 and 1.3429; *p* = 0.0069 and 0.0495).

According to the regression diagnostics of the two models (Figure S4), KEYNOTE (KN)‐010,^45^ KN‐048‐C (one of the trial arms using pembrolizumab as an add‐on to ChT for comparison with SOC in untreated HNSCC),^26^ KN‐181 (second‐line pembrolizumab versus ChT in esophageal cancer),^11^ and KN‐062‐P (paired arms comparing pembrolizumab monotherapy and SOC in untreated gastric cancer)^13^ were influential studies in the OS meta‐regression analysis, and KN‐590 (pembrolizumab as an add‐on to ChT compared with SOC in untreated esophageal cancer)^12^ and KN‐048‐C were influential studies in the PFS meta‐regression analysis. In KN‐010,^45^ patients with NSCLC harboring epidermal growth factor receptor (*EGFR*) mutations and disease progression after platinum‐based ChT and an *EGFR* tyrosine kinase inhibitor were eligible and constituted 8.8% of the patients in the pembrolizumab monotherapy arm and 7.6% of the patients in the docetaxel arm. Moreover, a meta‐analysis demonstrated that pembrolizumab significantly prolonged OS for patients with wild‐type *EGFR*, not mutant *EGFR*.^68^ Several studies have demonstrated that the frequency of driver mutations in lung adenocarcinoma varies by race and ethnicity, with *EGFR* mutations being the most prevalent in Asian patients.^69^ In KN‐181^70^ and KN‐590,^71^, ^72^ the histology of the Asian participants was primarily esophageal squamous cell carcinoma, consistent with the epidemiologic differences between Eastern and Western countries.^73^ Similar phenomena have been observed in HNSCC, which is characterized by regional heterogeneity in risk factors and anatomical sites. In Southeast Asia, a high prevalence of oral cavity cancer is associated with the consumption of areca nuts, whereas oropharyngeal human papillomavirus infection contributes to greater rates of oropharyngeal cancer in North America and Western Europe.^74^ Additionally, in KN‐062‐P, the proportions of Asian participants with ECOG PS scores of 0 and CPSs of 10 or greater were higher than those of the global population.^43^ Collectively, our findings highlight the unique features of diverse races and ethnicities and how different subpopulations may unequally benefit from pembrolizumab.

In RCTs, masking is crucial to ensure an unbiased estimate of the treatment effect; an open‐label design can introduce bias and exaggerate the results.^75^, ^76^ However, masking is only achievable in certain circumstances based on the nature of the experimental treatment. For instance, participants or investigators may be aware of the assigned treatment because some adverse events, such as immune‐related adverse events, could be indicative of ICI monotherapy. In this systematic review, 88.24% of the trials with pembrolizumab monotherapy arms (*m* = 17) used an open‐label design. The use of placebos may help reduce bias, but placebo production can be challenging and time‐consuming. The accrual rate may be influenced by the placebo design, and accrual projections are crucial, particularly if competitive studies are being conducted simultaneously. Consequently, objective and reliable outcome measurements, independent outcome adjudications (e.g., blinded independent central radiology review for PFS), and sensitivity analyses with censoring rules could be employed to minimize this risk. These practical issues are unavoidable in trial design. Further investigation is warranted to understand the relationship between an open‐label design and any observed differences between Asian and non‐Asian patients.

The strengths of this meta‐analysis include its rigorous data extraction and thorough meta‐regression analysis; these provide comprehensive assessments of the relative efficacy of pembrolizumab for Asian participants and insight into global trial design. These encouraging results may help promote the expansion of global trials in Asia. The inaccessibility of individual patient data is a major limitation, rendering comparisons of demographic and disease characteristics between subgroups challenging. Because of restricted data sources, the efficacy for Asian patients was not compared to other subdivided races and ethnicities. In the meta‐regression model for OS, the lower proportions of ECOG PS scores of 0 (≤50.28%) and men (≤81.92%) in the experimental arms resulted in superior OS benefits for the non‐Asian subgroups (estimated ratio of HRs, 1.5307 and 1.3214; *p* = 0.0085 and 0.0411, respectively). However, sex was discovered in another meta‐analysis not to be significantly associated with the efficacy of ICIs.^77^ Therefore, the two aforementioned covariates could be ascribed to other unidentified factors affecting the observed differences. Second, post‐study treatment, especially ICIs, could be a confounding source, but analyzing disparities in drug availability is beyond the scope of this study.^78^ The percentages of patients receiving any subsequent anticancer treatment or post‐progression PD‐(L)1 inhibitor (Table S5) had no significant effect on the results of the meta‐regression model for OS (Table S6). Finally, our attrition due to the exclusion of trials without available subgroup analyses may reduce the statistical credibility of our meta‐analysis. The inherent nature of study selection in this meta‐analytic study could partially explain the vulnerability of the pooled ratio of HRs for OS and the inconsistent results between OS and PFS.

## 5 CONCLUSIONS

This meta‐analysis of phase 3 RCTs investigating the efficacy of pembrolizumab in advanced cancers indicates that pembrolizumab may provide a greater OS benefit for Asian than non‐Asian patients.

## AUTHOR CONTRIBUTIONS

**Shang‐Hsuan Peng:** Data curation (lead); formal analysis (supporting); investigation (lead); software (supporting); validation (supporting); visualization (lead); writing – original draft (lead). **Ching‐Hung Lin:** Investigation (supporting); writing – review and editing (supporting). **I‐Chun Chen:** Investigation (supporting); writing – review and editing (supporting). **Ying‐Chun Shen:** Investigation (supporting); writing – review and editing (supporting). **Dwan‐Ying Chang:** Investigation (supporting); writing – review and editing (supporting). **Tom Wei‐Wu Chen:** Investigation (supporting); writing – review and editing (supporting). **Shu‐Min Huang:** Project administration (supporting). **Fu‐Chang Hu:** Formal analysis (lead); investigation (lead); methodology (lead); software (lead); visualization (lead); writing – review and editing (supporting). **Yen‐Shen Lu:** Conceptualization (lead); data curation (lead); formal analysis (lead); funding acquisition (lead); investigation (lead); methodology (lead); resources (lead); supervision (lead); validation (lead); writing – review and editing (lead).

## FUNDING INFORMATION

This work was supported by the National Science and Technology Council, Taiwan (MOST 110‐2314‐B‐002‐218).

## CONFLICT OF INTEREST STATEMENT

Ching‐Hung Lin reports honoraria from AstraZeneca, Eli Lilly, Novartis, and Pfizer, consulting for AstraZeneca, Eli Lilly, Novartis, and Pfizer. Tom Wei‐Wu Chen reports honoraria from AstraZeneca, Daiichi Sankyo, Eisai, Eli Lilly, Novartis, and Roche, consulting for AstraZeneca, Blueprint Medicines, Daiichi Sankyo, Eisai, and Roche, and research funding from Eisai and Epizyme. Yen‐Shen Lu reports honoraria from AstraZeneca, Daiichi Sankyo, Eisai, EuroPharma, Eli Lilly, Merck Sharp & Dohme, Novartis, Pfizer, and Roche, consulting for Eli Lilly, Novartis, Pfizer, and Roche, and research funding from AstraZeneca, Merck Sharp & Dohme, and Novartis. The remaining authors declare no conflicts of interest.

## ROLE OF THE FUNDER

The funder did not play a role in the design of the study; the collection, analysis, and interpretation of the data; the writing of the manuscript; and the decision to submit the manuscript for publication.

## DISCLAIMERS

The authors of this study are solely responsible for the interpretation and presentation of the data.

## PRIOR PRESENTATIONS

Results in this manuscript have been presented in part on a poster at the 2022 Taipei International Breast Cancer Symposium and selected for online publication abstract by the 2023 ASCO Annual Meeting.

## Supporting information


Appendix S1.

## Data Availability

The data underlying this article are available in the article and its supplementary material.
