# Supplementary material for: Disparity in survival benefits of pembrolizumab between Asian and non‐Asian patients with advanced cancers: A systematic review and meta‐regression analysis
Source: Cancer Med. 2023 Sep 22;12(19):20035–51. doi: 10.1002/cam4.6563 (PMC10587960; doi:10.1002/cam4.6563)
Supplement: Supplementary file 1 — Appendix S1. [file CAM4-12-20035-s001.docx]

Supplementary Tables and Figures

TABLE OF CONTENTS

[**Table 1.** Literature Search Strategies…………………………………………………………………………..P. 2](#TableS1)

[**Table 2.** Risk of Bias Assessments………………………………………………………………………….....P. 3](#TableS2)

[**Table 3.** Primary Endpoints and U.S. FDA Label Indications by PD-L1 Expression........................................P. 5](#TableS3)

[**Table 4.** Composition of Reported Asian and Non-Asian Subgroups in Selected Studies.................................P. 7](#TableS4)

[**Table 5.** Subsequent Therapy.............................................................................................................................P.1](#TableS7)5

[**Table 6.** Sensitivity Analyses.............................................................................................................................P.1](#TableS8)7

[**Figure 1**. Random-Effects Meta-analyses of Overall Survival and Progression-free Survival........................P.](#FigureS1) 20

[**Figure 2**. Normal *Q-Q* Plots of the Linear Fixed-Effects Meta-Regression Models........................................P.](#FigureS2) 22

[**Figure 3.** Funnel Plots of the Linear Fixed-Effects Meta-Regression Models.................................................P. 2](#FigureS3)4

[**Figure 4.** Influential Study Diagnostics for the Linear Fixed-Effects Meta-Regression Models.....................P. 2](#FigureS4)6

Table 1. Literature Search Strategies

| **Database** | **Search strategy** | **Results** | **Date** |
| --- | --- | --- | --- |
| PubMed | "pembrolizumab"[Supplementary Concept] OR "pembrolizumab"[All Fields]) AND (clinicaltrial[Filter] OR randomizedcontrolledtrial[Filter] | 563 | 2022/06/09 |
| Cochrane Library | Title, abstract, and keywords: “pembrolizumab”  Search limits: trials (content type); otherwise: default | 2307 | 2022/06/09 |
| Embase | “pembrolizumab” AND (“randomized controlled trial” OR “controlled clinical trial”) | 1746 | 2022/06/09 |
| Web of Science | ALL = (pembrolizumab) AND ALL = (randomized controlled trial) | 446 | 2022/06/09 |

**Table 2.** Risk of Bias Assessments

|  |  |  |  |  | **Domain*** | | | | |  |
| --- | --- | --- | --- | --- | --- | --- | --- | --- | --- | --- |
| **Trial**  **Name** | **Cancer Type** | **Setting** | **Experimental**  **Treatment** | **Control**  **Treatment** | **1** | **2** | **3** | **4** | **5** | **Overall Bias** |
| KN-010 | NSCLC | 2L | Monotherapy | ChT | Low | Low | Low | Low | Low | Low |
| KN-024 | NSCLC | 1L | Monotherapy | ChT | Low | Low | Low | Low | Low | Low |
| KN-042 | NSCLC | 1L | Monotherapy | ChT | Low | Some concerns^¶^ | Low | Low | Low | Some concerns |
| KN-189^†^ | NSCLC-ADC | 1L | Add-on to ChT | ChT | Low | Low | Low | Low | Low | Low |
| KN-407^†^ | NSCLC-SQ | 1L | Add-on to ChT | ChT | Low | Low | Low | Low | Low | Low |
| KN-240^†^ | HCC | 2L | Monotherapy | BSC | Low | Low | Low | Low | Low | Low |
| KN-394^†^ | HCC | 2L | Monotherapy | BSC | Low | Low | Low | Low | Low | Low |
| KN-040 | HNSCC | 2/3L | Monotherapy | ChT | Low | Low | Low | Low | Low | Low |
| KN-048-P | HNSCC | 1L | Monotherapy | ChT+cetuximab | Low | Some concerns^¶^ | Low | Low | Low | Some concerns |
| KN-048-C |  |  | Add-on to ChT |  |  |  |  |  |  |  |
| KN-181 | EC | 2L | Monotherapy | ChT | Low | Low | Low | Low | Low | Low |
| KN-590^†^ | EC | 1L | Add-on to ChT | ChT | Low | Low | Low | Low | Low | Low |
| KN-061 | GC | 2L | Monotherapy | ChT | Low | Low | Low | Low | Low | Low |
| KN-062-P | GC | 1L | Monotherapy | ChT | Low  Low | Low  Low | Low | Low | Low | Low |
| KN-062-C^†^ |  |  | Add-on to ChT |  |  |  | Low | Low | Low | Low |
| KN-119 | ABC | 2/3L | Monotherapy | ChT | Low | Low | Low | Low | Low | Low |
| KN-355^†^ | ABC | 1L | Add-on to ChT | ChT | Low | Low | Low | Low | Low | Low |
| KN-426 | ccRCC | 1L | Add-on to MKI | MKI | Low | Some concerns^¶^ | Low | Low | Low | Some concerns |
| KN-581 | ccRCC | 1L | Add-on to MKI | MKI | Low | Low | Low | Low | Low | Low |
| KN-006 | Melanoma | 1/2L | Monotherapy | Ipilimumab | Low | Low | Low | Low | Low | Low |
| KN-045 | UC | 2L | Monotherapy | ChT | Low | Low | Low | Low | Low | Low |
| KN-361-P | UC | 1L | Monotherapy | ChT | Low | Some concerns^¶^ | Low | Low | Low | Some concerns |
| KN-361-C |  |  | Add-on to ChT |  |  |  |  |  |  |  |
| KN-122 | NPC | 2/3L | Monotherapy | ChT | Low | Low | Low | Low | Low | Low |
| KN-177 | MSI-H CRC | 1L | Monotherapy | ChT+biologics | Low | Low | Low | Low | Low | Low |
| KN-604^†^ | SCLC | 1L | Add-on to ChT | ChT | Low | Low | Low | Low | Low | Low |
| KN-775 | EMC | 2/3L | Combination | ChT | Low | Low | Low | Low | Low | Low |
| KN-826^†^ | CC | 1L | Add-on to ChT | ChT±bevacizumab | Low | Low | Low | Low | Low | Low |
| ETOP 9-15 | PM | 2L | Monotherapy | ChT | Low | Low | Low | Low | Low | Low |

(The footnote is presented on page 4.)

Abbreviations: 1/2/3L, first/second/third-line; ABC, advanced breast cancer; ADC, adenocarcinoma; BSC, best supportive care; C, combination arm; CC, cervical cancer; ccRCC, clear cell renal cell carcinoma; ChT, chemotherapy; CRC, colorectal cancer; EC, esophageal cancer; EMC, endometrial cancer; ETOP, European Thoracic Oncology Platform; GC, gastric cancer; HCC, hepatocellular carcinoma; HNSCC, head and neck squamous cell carcinoma; KN, KEYNOTE; MKI, multikinase inhibitor; MSI-H, microsatellite instability-high; NPC, nasopharyngeal cancer; NSCLC, non-small cell lung cancer; P, pembrolizumab monotherapy arm; PM, pleural mesothelioma; SQ, squamous cell carcinoma; UC, urothelial carcinoma

^*^ The domains of risk-of-bias assessment include (1) randomization process, (2) deviations from intended interventions, (3) missing outcome data, (4) measurement of the outcome, and (5) selection of reported result.

^†^ Double-blind, placebo-controlled design (*n* = 9); KN-062 was a partially blinded trial.

^¶^ In the judgment of risk-of-bias for deviations from intended interventions, some concerns were mainly attributed to few patients (≤1% of the total population) receiving non-study drugs or prohibited medications in the trial context.

**Table 3.** Primary Endpoints and U.S. FDA Label Indications by PD-L1 Expression in Selected Studies

|  |  |  |  |  |  | **PD-L1 Expression** | | | |
| --- | --- | --- | --- | --- | --- | --- | --- | --- | --- |
| **Trial**  **Name** | **Cancer Type** | **Setting** | **Experimental**  **Treatment** | **Control**  **Treatment** | **Primary Endpoints** | **Inclusion Criteria** | **Hierarchical Testing*** | | **U.S. FDA**  **Label Indications** |
|  |  |  |  |  |  |  | **OS** | **PFS** |  |
| KN-010 | NSCLC | 2L | Monotherapy | ChT | PFS/OS | TPS1 | **TPS1** | **TPS50** | TPS1 |
| KN-024 | NSCLC | 1L | Monotherapy | ChT | PFS | TPS50 | **TPS50** | **TPS50** | TPS50 |
| KN-042 | NSCLC | 1L | Monotherapy | ChT | OS | TPS1 | **TPS1** | *NT* | TPS1 |
| KN-189 | NSCLC-ADC | 1L | Add-on to ChT | ChT | PFS/OS | All comers | **All comers** | **All comers** | All comers |
| KN-407 | NSCLC-SQ | 1L | Add-on to ChT | ChT | PFS/OS | All comers | **All comers** | **All comers** | All comers |
| KN-240 | HCC | 2L | Monotherapy | BSC | PFS/OS | All comers | *NS* | *NS* | All comers^†^ |
| KN-394 | HCC | 2L | Monotherapy | BSC | OS | All comers | **All comers** | **All comers** | —^‡^ |
| KN-040 | HNSCC | 2/3L | Monotherapy | ChT | OS | All comers | **All comers** | *NS* | All comers |
| KN-048-P | HNSCC | 1L | Monotherapy | ChT+cetuximab | PFS/OS | All comers | **All comers**^¶^ | *NS* | CPS1 |
| KN-048-C |  |  | Add-on to ChT |  |  |  | **All comers** | *NS* | All comers |
| KN-181 | EC | 2L | Monotherapy | ChT | OS | All comers | **CPS10**^§^ | *NT* | CPS10 (SQ)^§^ |
| KN-590 | EC | 1L | Add-on to ChT | ChT | PFS/OS | All comers | **All comers** | **All comers** | All comers |
| KN-061 | GC | 2L | Monotherapy | ChT | PFS/OS | All comers | *NS (CPS1)* | *NS (CPS1)* | — |
| KN-062-P | GC | 1L | Monotherapy | ChT | PFS/OS | CPS1 | **CPS1**^¶^ | *NT* | — |
| KN-062-C |  |  | Add-on to ChT |  |  |  | *NS* | *NS* | — |
| KN-119 | ABC | 2/3L | Monotherapy | ChT | OS | All comers | *NS (CPS10)* | *NT* | — |
| KN-355 | ABC | 1L | Add-on to ChT | ChT | PFS/OS | All comers | **CPS10** | **CPS10** | CPS10 |
| KN-426 | ccRCC | 1L | Add-on to MKI | MKI | PFS/OS | All comers | **All comers** | **All comers** | All comers |
| KN-581 | ccRCC | 1L | Add-on to MKI | MKI | PFS | All comers | **All comers** | **All comers** | All comers |
| KN-006 | Melanoma | 1/2L | Monotherapy | Ipilimumab | PFS/OS | All comers | **All comers** | **All comers** | All comers |
| KN-045 | UC | 2L | Monotherapy | ChT | PFS/OS | All comers | **All comers** | *NS* | All comers |
| KN-361-P | UC | 1L | Monotherapy | ChT | PFS/OS | All comers | *NT* | *NT* | — |
| KN-361-C |  |  | Add-on to ChT |  |  |  | *NS* | *NS* | — |
| KN-122 | NPC | 2/3L | Monotherapy | ChT | OS | All comers | *NS* | *NS* | — |
| KN-177 | MSI-H CRC | 1L | Monotherapy | ChT+biologics | PFS/OS | All comers | *NS* | **All comers** | All comers |
| KN-604 | SCLC | 1L | Add-on to ChT | ChT | PFS/OS | All comers | *NS* | **All comers** | — |
| KN-775 | EMC | 2/3L | Combination | ChT | PFS/OS | All comers | **All comers** | **All comers** | All comers^††^ |
| KN-826 | CC | 1L | Add-on to ChT | ChT±bevacizumab | PFS/OS | All comers | **All comers** | **All comers** | CPS1 |
| ETOP 9-15 | PM | 2L | Monotherapy | ChT | PFS | All comers | *NS* | *NS* | — |

(The footnote is presented on page 6.)

Abbreviations: 1/2/3L, first/second/third-line; ABC, advanced breast cancer; ADC, adenocarcinoma; BSC, best supportive care; C, combination arm; CC, cervical cancer; ccRCC, clear cell renal cell carcinoma; ChT, chemotherapy; CPS, combined positive score; CRC, colorectal cancer; EC, esophageal cancer; EMC, endometrial cancer; ETOP, European Thoracic Oncology Platform; GC, gastric cancer; HCC, hepatocellular carcinoma; HNSCC, head and neck squamous cell carcinoma; KN, KEYNOTE; MKI, multikinase inhibitor; MSI-H, microsatellite instability-high; NPC, nasopharyngeal cancer; *NS*, non-significant; *NT*, not tested; NSCLC, non-small cell lung cancer; OS, overall survival; P, pembrolizumab monotherapy arm; PD-L1, programmed death ligand-1; PFS, progression-free survival; PM, pleural mesothelioma; SQ, squamous cell carcinoma; TPS, tumor proportion score; UC, urothelial carcinoma; U.S FDA, the United States Food and Drug Administration

^*^ The endpoints met in superiority by hierarchical statistical testing are denoted by bold text.

^†^ The U.S. FDA granted accelerated approval only.

^¶^ The statistical criterion of non-inferiority was met.

^§^ In KN-181, the criterion of superiority was met in subsets with CPS ≥10, and the U.S. FDA granted regular approval in patients with esophageal squamous cell carcinoma and CPS ≥10.

^††^ The U.S. FDA approved pembrolizumab in combination with lenvatinib only for patients with mismatch repair (MMR)-proficient disease despite the endpoints met in all patients, including MMR-proficient and -deficient diseases.

^‡^ Pembrolizumab is not indicated in these settings, or the approval is not based on these trials.

**Table 4.** Composition of Reported Asian and Non-Asian Subgroups in Selected Studies (East Asia)

| **Trial**  **Name** | **Reported Subgroups** | | **East Asia** | | | | | | | | | | |
| --- | --- | --- | --- | --- | --- | --- | --- | --- | --- | --- | --- | --- | --- |
|  | **Asian** | **Non-Asian** | **Total** | **CN** | **HK** | **JP** | **SG** | **KR** | **TW** | **TH** | **MY** | **PH** | **VN** |
| **KN-010** | —^*^ | — | 3 | 0 | 0 | 1 | 0 | 1 | 1 | 0 | 0 | 0 | 0 |
| **KN-024** | JP | — | 1 | 0 | 0 | 1 | 0 | 0 | 0 | 0 | 0 | 0 | 0 |
| **KN-042** | — | — | 9 | 1 | 1 | 1 | 0 | 1 | 1 | 1 | 1 | 1 | 1 |
| KN-189 | JP | *N/A* | 1 | 0 | 0 | 1 | 0 | 0 | 0 | 0 | 0 | 0 | 0 |
| **KN-407** | — | — | 4 | 1 | 0 | 1 | 0 | 1 | 0 | 1 | 0 | 0 | 0 |
| **KN-240** | w/o JP | w/ JP | 6 | 0 | 1 | 1 | 0 | 1 | 1 | 1 | 0 | 1 | 0 |
| KN-394 | — | *N/A* | 5 | 1 | 1 | 0 | 0 | 1 | 1 | 0 | 1 | 0 | 0 |
| KN-040 | *N/A* | *N/A* | 1 | 0 | 0 | 0 | 0 | 1 | 0 | 0 | 0 | 0 | 0 |
| **KN-048-P** | — | — | 7 | 0 | 1 | 1 | 1 | 0 | 1 | 1 | 1 | 1 | 0  0 |
| **KN-048-C** |  |  |  |  |  |  |  |  |  |  |  |  |  |
| **KN-181** | — | — | 7 | 1 | 1 | 1 | 0 | 1 | 1 | 1 | 1 | 0 | 0 |
| **KN-590** | w/o TH/MY | w/ TH/MY | 7 | 1 | 1 | 1 | 0 | 1 | 1 | 1 | 1 | 0 | 0 |
| **KN-061** | — | NA, Europe, AU, IL | 6 | 0 | 1 | 1 | 1 | 1 | 1 | 0 | 1 | 0 | 0 |
| **KN-062-P** | — | NA, Europe, AU | 4 | 0 | 1 | 1 | 0 | 1 | 1 | 0 | 0 | 0 | 0 |
| **KN-062-C** |  |  |  |  |  |  |  |  |  |  |  |  |  |
| KN-119 | — | *N/A* | 8 | 0 | 1 | 1 | 1 | 1 | 1 | 1 | 1 | 1 | 0 |
| **KN-355** | — | NA, Europe, AU, NZ | 5 | 0 | 1 | 1 | 0 | 1 | 1 | 0 | 1 | 0 | 0 |
| **KN-426** | — | European Union | 3 | 0 | 0 | 1 | 0 | 1 | 1 | 0 | 0 | 0 | 0 |
| **KN-581** | — | NA, Western Europe | 2 | 0 | 0 | 1 | 0 | 1 | 0 | 0 | 0 | 0 | 0 |
| KN-006 | *N/A* | *N/A* | 0 | 0 | 0 | 0 | 0 | 0 | 0 | 0 | 0 | 0 | 0 |
| KN-045 | JP | *N/A* | 4 | 0 | 0 | 1 | 1 | 1 | 1 | 0 | 0 | 0 | 0 |
| KN-361-P | *N/A* | *N/A* | 4 | 0 | 0 | 1 | 0 | 1 | 1 | 1 | 0 | 0 | 0 |
| KN-361-C |  |  |  |  |  |  |  |  |  |  |  |  |  |
| **KN-122** | — | NA | 7 | 0 | 1 | 0 | 1 | 1 | 1 | 1 | 1 | 1 | 0 |
| **KN-177** | — | NA, Western Europe | 4 | 0 | 0 | 1 | 1 | 1 | 1 | 0 | 0 | 0 | 0 |
| **KN-604** | — | — | 3 | 0 | 0 | 1 | 0 | 1 | 1 | 0 | 0 | 0 | 0 |
| **KN-775** | Asian^†^ | White | 3 | 0 | 0 | 1 | 0 | 1 | 1 | 0 | 0 | 0 | 0 |
| KN-826 | *N/A* | *N/A* | 3 | 0 | 0 | 1 | 0 | 1 | 1 | 0 | 0 | 0 | 0 |
| ETOP 9-15 | *N/A* | *N/A* | 0 | 0 | 0 | 0 | 0 | 0 | 0 | 0 | 0 | 0 | 0 |
|  |  |  | Total | 5 | 11 | 21 | 6 | 21 | 19 | 9 | 9 | 5 | 1 |

(The footnote is presented on page 13.)

**Table 4.** Composition of Reported Asian and Non-Asian Subgroups in Selected Studies (North America and Europe)

| **Trial**  **Name** | **Reported Subgroups** | | **North America** | | | **Europe** | | | | | | | |
| --- | --- | --- | --- | --- | --- | --- | --- | --- | --- | --- | --- | --- | --- |
|  | **Asian** | **Non-Asian** | **Total** | **CA** | **US** | **Total** | **AT** | **BE** | **BG** | **CZ** | **DK** | **EE** | **FI** |
| **KN-010** | —^*^ | — | 2 | 1 | 1 | 14 | 0 | 1 | 0 | 1 | 1 | 0 | 0 |
| **KN-024** | JP | — | 2 | 1 | 1 | 10 | 1 | 1 | 0 | 0 | 0 | 0 | 0 |
| **KN-042** | — | — | 1 | 1 | 0 | 13 | 0 | 0 | 1 | 1 | 0 | 1 | 0 |
| KN-189 | JP | *N/A* | 2 | 1 | 1 | 11 | 1 | 1 | 0 | 0 | 1 | 0 | 1 |
| **KN-407** | — | — | 2 | 1 | 1 | 8 | 0 | 0 | 0 | 0 | 0 | 0 | 0 |
| **KN-240** | w/o JP | w/ JP | 2 | 1 | 1 | 11 | 0 | 1 | 0 | 0 | 1 | 0 | 0 |
| KN-394 | — | *N/A* | 0 | 0 | 0 | 0 | 0 | 0 | 0 | 0 | 0 | 0 | 0 |
| KN-040 | *N/A* | *N/A* | 2 | 1 | 1 | 15 | 0 | 1 | 0 | 0 | 0 | 0 | 0 |
| **KN-048-P** | — | — | 2 | 1 | 1 | 18 | 1 | 0 | 0 | 1 | 1 | 1 | 1 |
| **KN-048-C** |  |  |  |  |  |  |  |  |  |  |  |  |  |
| **KN-181** | — | — | 2 | 1 | 1 | 15 | 0 | 0 | 0 | 1 | 1 | 1 | 1 |
| **KN-590** | w/o TH/MY | w/ TH/MY | 2 | 1 | 1 | 7 | 0 | 0 | 0 | 0 | 1 | 0 | 0 |
| **KN-061** | — | NA, Europe, AU, IL | 2 | 1 | 1 | 12 | 0 | 1 | 0 | 0 | 1 | 1 | 1 |
| **KN-062-P** | — | NA, Europe, AU | 1 | 0 | 1 | 14 | 1 | 1 | 0 | 1 | 0 | 0 | 0 |
| **KN-062-C** |  |  |  |  |  |  |  |  |  |  |  |  |  |
| KN-119 | — | *N/A* | 1 | 0 | 1 | 12 | 0 | 1 | 0 | 0 | 0 | 0 | 0 |
| **KN-355** | — | NA, Europe, AU, NZ | 2 | 1 | 1 | 14 | 0 | 1 | 0 | 1 | 1 | 0 | 0 |
| **KN-426** | — | European Union | 2 | 1 | 1 | 10 | 0 | 0 | 0 | 1 | 0 | 0 | 0 |
| **KN-581** | — | NA, Western Europe | 2 | 1 | 1 | 14 | 1 | 1 | 0 | 1 | 0 | 0 | 0 |
| KN-006 | *N/A* | *N/A* | 2 | 1 | 1 | 9 | 1 | 1 | 0 | 0 | 0 | 0 | 0 |
| KN-045 | JP | *N/A* | 2 | 1 | 1 | 16 | 1 | 1 | 0 | 0 | 1 | 0 | 0 |
| KN-361-P | *N/A* | *N/A* | 2 | 1 | 1 | 9 | 0 | 1 | 0 | 0 | 0 | 0 | 0 |
| KN-361-C |  |  |  |  |  |  |  |  |  |  |  |  |  |
| **KN-122** | — | NA | 2 | 1 | 1 | 0 | 0 | 0 | 0 | 0 | 0 | 0 | 0 |
| **KN-177** | — | NA, Western Europe | 2 | 1 | 1 | 13 | 0 | 1 | 0 | 0 | 1 | 0 | 1 |
| **KN-604** | — | — | 2 | 1 | 1 | 9 | 0 | 0 | 0 | 0 | 0 | 0 | 0 |
| **KN-775** | Asian^†^ | White | 2 | 1 | 1 | 8 | 0 | 0 | 0 | 0 | 0 | 0 | 0 |
| KN-826 | *N/A* | *N/A* | 2 | 1 | 1 | 6 | 0 | 0 | 0 | 0 | 0 | 0 | 0 |
| ETOP 9-15 | *N/A* | *N/A* | 0 | 0 | 0 | 3 | 0 | 0 | 0 | 0 | 0 | 0 | 0 |
|  |  |  | Total | 22 | 23 | Total | 7 | 14 | 1 | 8 | 10 | 4 | 5 |

(The footnote is presented on page 13.)

**Table 4.** Composition of Reported Asian and Non-Asian Subgroups in Selected Studies (Europe)

| **Trial**  **Name** | **Reported Subgroups** | | **Europe** | | | | | | | | | | |
| --- | --- | --- | --- | --- | --- | --- | --- | --- | --- | --- | --- | --- | --- |
|  | **Asian** | **Non-Asian** | **FR** | **DE** | **GR** | **HU** | **IE** | **IT** | **LV** | **LT** | **NL** | **NO** | **PL** |
| **KN-010** | —^*^ | — | 1 | 1 | 1 | 1 | 0 | 1 | 0 | 1 | 1 | 0 | 0 |
| **KN-024** | JP | — | 1 | 1 | 0 | 1 | 1 | 1 | 0 | 0 | 1 | 0 | 0 |
| **KN-042** | — | — | 0 | 0 | 0 | 1 | 0 | 0 | 1 | 1 | 0 | 0 | 1 |
| KN-189 | JP | *N/A* | 1 | 1 | 0 | 0 | 1 | 1 | 0 | 0 | 1 | 0 | 0 |
| **KN-407** | — | — | 1 | 1 | 0 | 1 | 0 | 1 | 0 | 0 | 1 | 0 | 1 |
| **KN-240** | w/o JP | w/ JP | 1 | 1 | 0 | 1 | 1 | 1 | 0 | 0 | 0 | 1 | 1 |
| KN-394 | — | *N/A* | 0 | 0 | 0 | 0 | 0 | 0 | 0 | 0 | 0 | 0 | 0 |
| KN-040 | *N/A* | *N/A* | 1 | 1 | 0 | 1 | 1 | 1 | 0 | 1 | 1 | 0 | 1 |
| **KN-048-P** | — | — | 0 | 1 | 1 | 1 | 0 | 1 | 1 | 0 | 1 | 1 | 1 |
| **KN-048-C** |  |  |  |  |  |  |  |  |  |  |  |  |  |
| **KN-181** | — | — | 1 | 1 | 0 | 0 | 1 | 1 | 0 | 0 | 1 | 1 | 0 |
| **KN-590** | w/o TH/MY | w/ TH/MY | 1 | 1 | 0 | 0 | 0 | 0 | 0 | 0 | 0 | 0 | 0 |
| **KN-061** | — | NA, Europe, AU, IL | 0 | 1 | 0 | 0 | 1 | 1 | 0 | 0 | 0 | 1 | 1 |
| **KN-062-P** | — | NA, Europe, AU | 0 | 1 | 0 | 1 | 0 | 1 | 1 | 1 | 1 | 0 | 1 |
| **KN-062-C** |  |  |  |  |  |  |  |  |  |  |  |  |  |
| KN-119 | — | *N/A* | 1 | 1 | 0 | 0 | 1 | 1 | 0 | 0 | 1 | 0 | 1 |
| **KN-355** | — | NA, Europe, AU, NZ | 1 | 1 | 0 | 1 | 1 | 1 | 0 | 0 | 1 | 0 | 1 |
| **KN-426** | — | European Union | 1 | 1 | 0 | 1 | 1 | 0 | 0 | 0 | 0 | 0 | 1 |
| **KN-581** | — | NA, Western Europe | 1 | 1 | 1 | 0 | 1 | 1 | 0 | 0 | 1 | 0 | 1 |
| KN-006 | *N/A* | *N/A* | 1 | 1 | 0 | 0 | 0 | 0 | 0 | 0 | 1 | 1 | 0 |
| KN-045 | JP | *N/A* | 1 | 1 | 0 | 1 | 1 | 1 | 0 | 0 | 1 | 1 | 1 |
| KN-361-P | *N/A* | *N/A* | 1 | 1 | 0 | 1 | 1 | 0 | 0 | 0 | 1 | 0 | 0 |
| KN-361-C |  |  |  |  |  |  |  |  |  |  |  |  |  |
| **KN-122** | — | NA | 0 | 0 | 0 | 0 | 0 | 0 | 0 | 0 | 0 | 0 | 0 |
| **KN-177** | — | NA, Western Europe | 1 | 1 | 0 | 0 | 1 | 1 | 0 | 0 | 1 | 1 | 0 |
| **KN-604** | — | — | 1 | 1 | 0 | 1 | 1 | 0 | 0 | 0 | 0 | 0 | 1 |
| **KN-775** | Asian^†^ | White | 1 | 1 | 0 | 0 | 1 | 1 | 0 | 0 | 0 | 0 | 1 |
| KN-826 | *N/A* | *N/A* | 1 | 1 | 0 | 0 | 0 | 1 | 0 | 0 | 0 | 0 | 0 |
| ETOP 9-15 | *N/A* | *N/A* | 0 | 0 | 0 | 0 | 0 | 0 | 0 | 0 | 0 | 0 | 0 |
|  |  | Total | 19 | 22 | 3 | 13 | 15 | 17 | 3 | 4 | 15 | 7 | 14 |

(The footnote is presented on page 13.)

**Table 4.** Composition of Reported Asian and Non-Asian Subgroups in Selected Studies (Europe)

| **Trial**  **Name** | **Reported Subgroups** | | **Europe** | | | | | | | |
| --- | --- | --- | --- | --- | --- | --- | --- | --- | --- | --- |
|  | **Asian** | **Non-Asian** | **PT** | **RO** | **RU** | **ES** | **SE** | **CH** | **UA** | **UK** |
| **KN-010** | —^*^ | — | 1 | 0 | 1 | 1 | 0 | 0 | 0 | 1 |
| **KN-024** | JP | — | 0 | 0 | 0 | 1 | 0 | 0 | 0 | 1 |
| **KN-042** | — | — | 1 | 1 | 1 | 0 | 1 | 1 | 1 | 0 |
| KN-189 | JP | *N/A* | 0 | 0 | 0 | 1 | 0 | 0 | 0 | 1 |
| **KN-407** | — | — | 0 | 0 | 1 | 1 | 0 | 0 | 0 | 0 |
| **KN-240** | w/o JP | w/ JP | 0 | 0 | 1 | 0 | 0 | 0 | 0 | 1 |
| KN-394 | — | *N/A* | 0 | 0 | 0 | 0 | 0 | 0 | 0 | 0 |
| KN-040 | *N/A* | *N/A* | 1 | 0 | 1 | 1 | 1 | 1 | 0 | 1 |
| **KN-048-P** | — | — | 0 | 0 | 1 | 1 | 1 | 1 | 0 | 1 |
| **KN-048-C** |  |  |  |  |  |  |  |  |  |  |
| **KN-181** | — | — | 1 | 0 | 1 | 1 | 1 | 0 | 0 | 1 |
| **KN-590** | w/o TH/MY | w/ TH/MY | 0 | 1 | 1 | 1 | 0 | 0 | 0 | 1 |
| **KN-061** | — | NA, Europe, AU, IL | 0 | 0 | 1 | 1 | 0 | 0 | 0 | 1 |
| **KN-062-P** | — | NA, Europe, AU | 0 | 0 | 1 | 1 | 0 | 1 | 0 | 1 |
| **KN-062-C** |  |  |  |  |  |  |  |  |  |  |
| KN-119 | — | *N/A* | 0 | 0 | 1 | 1 | 1 | 1 | 0 | 1 |
| **KN-355** | — | NA, Europe, AU, NZ | 0 | 0 | 1 | 1 | 0 | 0 | 1 | 1 |
| **KN-426** | — | European Union | 0 | 0 | 1 | 1 | 0 | 0 | 1 | 1 |
| **KN-581** | — | NA, Western Europe | 0 | 0 | 1 | 1 | 0 | 1 | 0 | 1 |
| KN-006 | *N/A* | *N/A* | 0 | 0 | 0 | 1 | 1 | 0 | 0 | 1 |
| KN-045 | JP | *N/A* | 1 | 1 | 0 | 1 | 1 | 0 | 0 | 1 |
| KN-361-P | *N/A* | *N/A* | 0 | 0 | 1 | 1 | 0 | 0 | 0 | 1 |
| KN-361-C |  |  |  |  |  |  |  |  |  |  |
| **KN-122** | — | NA | 0 | 0 | 0 | 0 | 0 | 0 | 0 | 0 |
| **KN-177** | — | NA, Western Europe | 0 | 0 | 0 | 1 | 1 | 1 | 0 | 1 |
| **KN-604** | — | — | 0 | 0 | 1 | 1 | 0 | 1 | 0 | 1 |
| **KN-775** | Asian^†^ | White | 0 | 0 | 1 | 1 | 0 | 0 | 0 | 1 |
| KN-826 | *N/A* | *N/A* | 0 | 0 | 1 | 1 | 0 | 0 | 1 | 0 |
| ETOP 9-15 | *N/A* | *N/A* | 0 | 0 | 0 | 1 | 0 | 1 | 0 | 1 |
|  |  | Total | 5 | 3 | 18 | 22 | 8 | 9 | 4 | 21 |

(The footnote is presented on page 13.)

**Table 4.** Composition of Reported Asian and Non-Asian Subgroups in Selected Studies (Oceania, Africa, and Middle East)

| **Trial**  **Name** | **Reported Subgroups** | | **Oceania** | | | **Africa** | **Middle East** | | |
| --- | --- | --- | --- | --- | --- | --- | --- | --- | --- |
|  | **Asian** | **Non-Asian** | **Total** | **AU** | **NZ** | **ZA** | **Total** | **IL** | **TR** |
| **KN-010** | —^*^ | — | 1 | 1 | 0 | 1 | 0 | 0 | 0 |
| **KN-024** | JP | — | 2 | 1 | 1 | 0 | 1 | 1 | 0 |
| **KN-042** | — | — | 0 | 0 | 0 | 1 | 1 | 0 | 1 |
| KN-189 | JP | *N/A* | 1 | 1 | 0 | 0 | 1 | 1 | 0 |
| **KN-407** | — | — | 1 | 1 | 0 | 0 | 1 | 0 | 1 |
| **KN-240** | w/o JP | w/ JP | 1 | 1 | 0 | 0 | 2 | 1 | 1 |
| KN-394 | — | *N/A* | 0 | 0 | 0 | 0 | 0 | 0 | 0 |
| KN-040 | *N/A* | *N/A* | 1 | 1 | 0 | 0 | 0 | 0 | 0 |
| **KN-048-P** | — | — | 1 | 1 | 0 | 1 | 2 | 1 | 1 |
| **KN-048-C** |  |  |  |  |  |  |  |  |  |
| **KN-181** | — | — | 1 | 1 | 0 | 0 | 2 | 1 | 1 |
| **KN-590** | w/o TH/MY | w/ TH/MY | 1 | 1 | 0 | 1 | 1 | 0 | 1 |
| **KN-061** | — | NA, Europe, AU, IL | 2 | 1 | 1 | 1 | 2 | 1 | 1 |
| **KN-062-P** | — | NA, Europe, AU | 2 | 1 | 1 | 1 | 0 | 0 | 0 |
| **KN-062-C** |  |  |  |  |  |  |  |  |  |
| KN-119 | — | *N/A* | 2 | 1 | 1 | 1 | 1 | 0 | 1 |
| **KN-355** | — | NA, Europe, AU, NZ | 2 | 1 | 1 | 0 | 1 | 0 | 1 |
| **KN-426** | — | European Union | 0 | 0 | 0 | 0 | 0 | 0 | 0 |
| **KN-581** | — | NA, Western Europe | 1 | 1 | 0 | 0 | 1 | 1 | 0 |
| KN-006 | *N/A* | *N/A* | 2 | 1 | 1 | 0 | 1 | 1 | 0 |
| KN-045 | JP | *N/A* | 2 | 1 | 1 | 0 | 2 | 1 | 1 |
| KN-361-P | *N/A* | *N/A* | 0 | 0 | 0 | 1 | 2 | 1 | 1 |
| KN-361-C |  |  |  |  |  |  |  |  |  |
| **KN-122** | — | NA | 0 | 0 | 0 | 0 | 0 | 0 | 0 |
| **KN-177** | — | NA, Western Europe | 1 | 1 | 0 | 1 | 1 | 1 | 0 |
| **KN-604** | — | — | 1 | 1 | 0 | 0 | 2 | 1 | 1 |
| **KN-775** | Asian^†^ | White | 2 | 1 | 1 | 0 | 2 | 1 | 1 |
| KN-826 | *N/A* | *N/A* | 1 | 1 | 0 | 0 | 2 | 1 | 1 |
| ETOP 9-15 | *N/A* | *N/A* | 0 | 0 | 0 | 0 | 0 | 0 | 0 |
|  |  |  | Total | 20 | 8 | 9 | Total | 14 | 14 |

(The footnote is presented on page 13.)

**Table 4.** Composition of Reported Asian and Non-Asian Subgroups in Selected Studies (Latin America)

| **Trial**  **Name** | **Reported Subgroups** | | **Latin America** | | | | | | | | | |
| --- | --- | --- | --- | --- | --- | --- | --- | --- | --- | --- | --- | --- |
|  | **Asian** | **Non-Asian** | **Total** | **AR** | **BR** | **CL** | **CO** | **CR** | **GT** | **MX** | **PE** | **PR** |
| **KN-010** | —^*^ | — | 3 | 1 | 1 | 1 | 0 | 0 | 0 | 0 | 0 | 0 |
| **KN-024** | JP | — | 0 | 0 | 0 | 0 | 0 | 0 | 0 | 0 | 0 | 0 |
| **KN-042** | — | — | 7 | 1 | 1 | 1 | 1 | 0 | 1 | 1 | 1 | 0 |
| KN-189 | JP | *N/A* | 0 | 0 | 0 | 0 | 0 | 0 | 0 | 0 | 0 | 0 |
| **KN-407** | — | — | 1 | 0 | 0 | 0 | 0 | 0 | 0 | 1 | 0 | 0 |
| **KN-240** | w/o JP | w/ JP | 5 | 1 | 0 | 1 | 1 | 0 | 0 | 1 | 0 | 1 |
| KN-394 | — | *N/A* | 0 | 0 | 0 | 0 | 0 | 0 | 0 | 0 | 0 | 0 |
| KN-040 | *N/A* | *N/A* | 1 | 0 | 0 | 0 | 0 | 0 | 0 | 1 | 0 | 0 |
| **KN-048-P** | — | — | 6 | 1 | 1 | 1 | 1 | 0 | 0 | 1 | 1 | 0 |
| **KN-048-C** |  |  |  |  |  |  |  |  |  |  |  |  |
| **KN-181** | — | — | 5 | 1 | 1 | 0 | 1 | 0 | 0 | 1 | 1 | 0 |
| **KN-590** | w/o TH/MY | w/ TH/MY | 7 | 1 | 1 | 1 | 1 | 1 | 1 | 0 | 1 | 0 |
| **KN-061** | — | NA, Europe, AU, IL | 5 | 1 | 0 | 1 | 1 | 0 | 1 | 1 | 0 | 0 |
| **KN-062-P** | — | NA, Europe, AU | 7 | 1 | 1 | 1 | 1 | 0 | 1 | 1 | 0 | 1 |
| **KN-062-C** |  |  |  |  |  |  |  |  |  |  |  |  |
| KN-119 | — | *N/A* | 6 | 1 | 1 | 0 | 1 | 0 | 1 | 1 | 1 | 0 |
| **KN-355** | — | NA, Europe, AU, NZ | 5 | 1 | 1 | 1 | 1 | 0 | 0 | 1 | 0 | 0 |
| **KN-426** | — | European Union | 1 | 0 | 1 | 0 | 0 | 0 | 0 | 0 | 0 | 0 |
| **KN-581** | — | NA, Western Europe | 0 | 0 | 0 | 0 | 0 | 0 | 0 | 0 | 0 | 0 |
| KN-006 | *N/A* | *N/A* | 2 | 0 | 0 | 1 | 1 | 0 | 0 | 0 | 0 | 0 |
| KN-045 | JP | *N/A* | 3 | 0 | 0 | 1 | 0 | 0 | 0 | 0 | 1 | 1 |
| KN-361-P | *N/A* | *N/A* | 3 | 1 | 1 | 1 | 0 | 0 | 0 | 0 | 0 | 0 |
| KN-361-C |  |  |  |  |  |  |  |  |  |  |  |  |
| **KN-122** | — | NA | 0 | 0 | 0 | 0 | 0 | 0 | 0 | 0 | 0 | 0 |
| **KN-177** | — | NA, Western Europe | 1 | 0 | 1 | 0 | 0 | 0 | 0 | 0 | 0 | 0 |
| **KN-604** | — | — | 1 | 0 | 0 | 1 | 0 | 0 | 0 | 0 | 0 | 0 |
| **KN-775** | Asian^†^ | White | 4 | 1 | 1 | 0 | 1 | 0 | 0 | 1 | 0 | 0 |
| KN-826 | *N/A* | *N/A* | 5 | 1 | 0 | 1 | 1 | 0 | 0 | 1 | 1 | 0 |
| ETOP 9-15 | *N/A* | *N/A* | 0 | 0 | 0 | 0 | 0 | 0 | 0 | 0 | 0 | 0 |
|  |  |  | Total | 13 | 12 | 13 | 12 | 1 | 5 | 12 | 7 | 3 |

(The footnote is presented on page 13.)

Abbreviations: C, combination arm; ETOP, European Thoracic Oncology Platform; KN, KEYNOTE; P, pembrolizumab monotherapy arm; NA, North America; *N/A*, not available; w/, with; w/o, without. Country abbreviations are specified on page 14.

Selected studies with fully reported hazard ratios (HRs) for **OS** in both subgroups are denoted by **bold** text in the Trial Name column (*n* = 17, *m* = 19), among which those with HRs for **PFS** in both subgroups are denoted by underlining (*n* = 11, *m* = 12).

^*^ The reported Asian and non-Asian subgroups are defined as those patients enrolled from countries in and not in East Asia, unless other specified.

^†^ In KN-775, the reported subgroups were stratified by race.

Country Abbreviations

| Region | Abbreviations | Country | Region | Abbreviations | Country |
| --- | --- | --- | --- | --- | --- |
| East Asia | CN | China | Europe (continued) | LT | Lithuania |
|  | HK | Hong Kong |  | NL | Netherlands |
|  | JP | Japan |  | NO | Norway |
|  | SG | Singapore |  | PL | Poland |
|  | KR | South Korea |  | PT | Portugal |
|  | TW | Taiwan |  | RO | Romania |
|  | TH | Thailand |  | RU | Russia |
|  | MY | Malaysia |  | ES | Spain |
|  | PH | Philippines |  | SE | Sweden |
|  | VN | Vietnam |  | CH | Switzerland |
| North America | CA | Canada |  | UA | Ukraine |
|  | US | United States |  | UK | United Kingdom |
| Europe | AT | Austria | Oceania | AU | Australia |
|  | BE | Belgium |  | NZ | New Zealand |
|  | BG | Bulgaria | Africa | ZA | South Africa |
|  | CZ | Czech | Middle East | IL | Israel |
|  | DK | Denmark |  | TR | Turkey |
|  | EE | Estonia | Latin America | AR | Argentina |
|  | FI | Finland |  | BR | Brazil |
|  | FR | France |  | CL | Chile |
|  | DE | Germany |  | CO | Colombia |
|  | GR | Greece |  | CR | Costa Rica |
|  | HU | Hungary |  | GT | Guatemala |
|  | IE | Ireland |  | MX | Mexico |
|  | IT | Italy |  | PE | Peru |
|  | LV | Latvia |  | PR | Puerto Rico |

**Table 5.** Percentage of Any Subsequent Anticancer Therapy and Subsequent PD-(L)1 Inhibitor in Selected Studies

|  |  |  |  |  |  | **Experimental Arm** | | **Control Arm** | |
| --- | --- | --- | --- | --- | --- | --- | --- | --- | --- |
| **Trial**  **Name** | **Cancer Type** | **Setting** | **Experimental**  **Treatment** | **Control**  **Treatment** | **In-Study**  **Crossover** | **Any**  **(%)** | **PD-(L)1 Inhibitor**  **(%)** | **Any**  **(%)** | **PD-(L)1 Inhibitor**  **(%)** |
| **KN-010** | NSCLC | 2L | Monotherapy | ChT | Permitted | *N/A* | *N/A* | *N/A* | 22.16 |
| **KN-024** | NSCLC | 1L | Monotherapy | ChT | Permitted | 36.36 | 5.19 | *N/A* | 54.30 |
| **KN-042** | NSCLC | 1L | Monotherapy | ChT | Not permitted | 37.68 | 2.98 | 44.27 | 19.78 |
| KN-189 | NSCLC-ADC | 1L | Add-on to ChT | ChT | Permitted | 49.51 | 18.05 | 61.65 | 55.83 |
| **KN-407** | NSCLC-SQ | 1L | Add-on to ChT | ChT | Permitted | *N/A* | 4.32 | *N/A* | 31.67 |
| **KN-240** | HCC | 2L | Monotherapy | BSC | Not mentioned | 41.73 | 6.83 | 47.41 | 10.37 |
| KN-394 | HCC | 2L | Monotherapy | BSC | Not mentioned | 50.67 | 20.67 | 66.67 | 28.10 |
| KN-040 | HNSCC | 2/3L | Monotherapy | ChT | Not permitted | 34.01 | ^*^4.45 | 40.73 | ^*^12.50 |
| **KN-048-P** | HNSCC | 1L | Monotherapy | ChT+cetuximab | Not mentioned | 46.51 | 4.32 | 52.00 | 24.00 |
| **KN-048-C** |  |  | Add-on to ChT |  |  | 38.08 | 4.98 | *N/A* | *N/A* |
| **KN-181** | EC | 2L | Monotherapy | ChT | Protocol unavailable | *N/A* | 0.32 | *N/A* | 9.55 |
| **KN-590** | EC | 1L | Add-on to ChT | ChT | Not permitted | 43.16 | ^*^5.90 | 47.07 | ^*^9.31 |
| **KN-061** | GC | 2L | Monotherapy | ChT | Not mentioned | *N/A* | *N/A* | *N/A* | *N/A* |
| **KN-062-P** | GC | 1L | Monotherapy | ChT | Not mentioned | 52.34 | 4.69 | 52.80 | 13.20 |
| **KN-062-C** |  |  | Add-on to ChT |  |  | 45.91 | 4.28 |  |  |
| KN-119 | ABC | 2/3L | Monotherapy | ChT | Not permitted | *N/A* | *N/A* | *N/A* | *N/A* |
| **KN-355** | ABC | 1L | Add-on to ChT | ChT | Not permitted | *N/A* | *N/A* | *N/A* | *N/A* |
| **KN-426** | ccRCC | 1L | Add-on to MKI | MKI | Not mentioned | 39.35 | 5.79 | 56.41 | 39.39 |
| **KN-581** | ccRCC | 1L | Add-on to MKI | MKI | Not mentioned | 32.96 | ^*^9.86 | 57.70 | ^*^48.18 |
| KN-006 | Melanoma | 1/2L | Monotherapy | Ipilimumab | Not mentioned | 39.39 | 4.14 | 47.84 | 28.42 |
| KN-045 | UC | 2L | Monotherapy | ChT | Permitted | *N/A* | *N/A* | *N/A* | 9.56 |
| KN-361-P | UC | 1L | Monotherapy | ChT | Not mentioned | 41.04 | 4.56 | 61.08 | 48.01 |
| KN-361-C |  |  | Add-on to ChT |  |  | 35.33 | 6.55 |  |  |
| **KN-122** | NPC | 2/3L | Monotherapy | ChT | Protocol unavailable | 66.67 | ^†^5.98 | 61.21 | ^†^30.17 |
| **KN-177** | MSI-H CRC | 1L | Monotherapy | ChT+biologics | Permitted | 33.99 | 9.15 | 78.57 | 60.39 |
| **KN-604** | SCLC | 1L | Add-on to ChT | ChT | Not mentioned | 51.75 | ^*^3.95 | 64.89 | ^*^13.78 |
| **KN-775** | EMC | 2/3L | Combination | ChT | Not permitted | 27.98 | *N/A* | 48.08 | 10.34 |
| KN-826 | CC | 1L | Add-on to ChT | ChT±bevacizumab | Not mentioned | *N/A* | *N/A* | *N/A* | *N/A* |
| ETOP 9-15 | PM | 2L | Monotherapy | ChT | Permitted | *N/A* | *N/A* | *N/A* | 63.38 |

(The footnote is presented on page 18.)

Abbreviations: 1/2/3L, first/second/third-line; ABC, advanced breast cancer; ADC, adenocarcinoma; BSC, best supportive care; C, combination arm; CC, cervical cancer; ccRCC, clear cell renal cell carcinoma; ChT, chemotherapy; CRC, colorectal cancer; EC, esophageal cancer; EMC, endometrial cancer; ETOP, European Thoracic Oncology Platform; GC, gastric cancer; HCC, hepatocellular carcinoma; HNSCC, head and neck squamous cell carcinoma; KN, KEYNOTE; MKI, multikinase inhibitor; MSI-H, microsatellite instability-high; *N/A*, not available; NPC, nasopharyngeal cancer; NSCLC, non-small cell lung cancer; P, pembrolizumab monotherapy arm; PD-(L)1, programmed cell death (ligand)-1; PM, pleural mesothelioma; SQ, squamous cell carcinoma; UC, urothelial carcinoma

Selected studies with fully reported hazard ratios for **OS** in both subgroups are denoted by **bold** text in the Trial Name column (*n* = 17, *m* = 19).

^*^ Including immunotherapy other than PD-(L)1 inhibitors.

^†^ Total number of therapies, not number of patients.

**Table 6A.** Sensitivity Analyses for Linear Fixed-Effects Meta-Regression Analysis of Mean Differences in Natural Logarithms of Hazard Ratios for Overall Survival Between Asian and Non-Asian Subgroups

| **Covariates** | **Regression Coefficient Estimate** | **Standard Error** | ***Z* Value** | ***P* Value** | **Estimated Ratio of**  **Hazard Ratios**  **(95% CI)** |
| --- | --- | --- | --- | --- | --- |
| **A1. Any Subsequent Anticancer Therapy (*m* = 12)**^*^ | | | | | |
| Intercept | −0.4170 | 0.7171 | −0.5815 | .5609 | 0.6590 (0.1616-2.6869) |
| Add-on (to ChT or MKI) or combination with open-label design | 0.5646 | 0.3700 | 1.5260 | .1270 | 1.7587 (0.8517−3.6317) |
| ECOG PS scores of 0 ≤50.28% (experimental arm) | 0.4272 | 0.2443 | 1.7489 | .0803 | 1.5330 (0.9497−2.4744) |
| Men ≤81.92% (experimental arm) | 0.1566 | 0.1970 | 0.7950 | .4266 | 1.1695 (0.7949−1.7208) |
| Percentage of any subsequent anticancer therapy (control arm) | −0.0036 | 0.0130 | −0.2770 | .7818 | 0.9964 (0.9713-1.0221) |
| Percentage of any subsequent anticancer therapy (experimental arm) | −0.0013 | 0.0154 | −0.0848 | .9324 | 0.9987 (0.9689-1.0293) |
| **A2. Any Subsequent Anticancer Therapy (*m* = 12)**^**^ | | | | | |
| Intercept | −0.6576 | 0.2583 | −2.5457 | .0109 | 0.5181 (0.3123−0.8596) |
| Add-on (to ChT or MKI) or combination with open-label design | 0.5800 | 0.2682 | 2.1630 | .0305 | 1.7860 (1.0560−3.0210) |
| ECOG PS scores of 0 ≤50.28% (experimental arm) | 0.4279 | 0.2225 | 1.9231 | .0545 | 1.5340 (0.9918−2.3729) |
| Men ≤81.92% (experimental arm) | 0.1533 | 0.1773 | 0.8644 | .3874 | 1.1657 (0.8235−1.6499) |

Abbreviations: ChT, chemotherapy; CI, confidence interval; ECOG PS, Eastern Cooperative Oncology Group performance status; MKI, multikinase inhibitor

These meta-regression analyses were performed using the escalc() and rma() functions in the metafor package (version 3.8-1) of R 4.2.1.

^*^ The test for residual heterogeneity showed a χ^2^ statistic (df = 6) = 0.51, *P* = 1.00 and *I*^2^ ≈ 0%. Thus, the linear fixed-effects meta-regression model was reported.

^**^ The test for residual heterogeneity showed a χ^2^ statistic (df = 8) = 0.68, *P* = 1.00 and *I*^2^ ≈ 0%. Thus, the linear fixed-effects meta-regression model was reported.

**Table 6B.** Sensitivity Analyses for Linear Fixed-Effects Meta-Regression Analysis of Mean Differences in Natural Logarithms of Hazard Ratios for Overall Survival Between Asian and Non-Asian Subgroups

| **Covariates** | **Regression Coefficient Estimate** | **Standard Error** | ***Z* Value** | ***P* Value** | **Estimated Ratio of**  **Hazard Ratios**  **(95% CI)** |
| --- | --- | --- | --- | --- | --- |
| **B1. Subsequent PD-(L)1 Inhibitor (*m* = 14)**^†^ | | | | | |
| Intercept | −0.8615 | 0.3107 | −2.7724 | .0056 | 0.4354 (0.2298−0.7769) |
| Add-on (to ChT or MKI) or combination with open-label design | 0.7316 | 0.4209 | 1.7385 | .0821 | 2.0784 (0.9110−4.7422) |
| ECOG PS scores of 0 ≤50.28% (experimental arm) | 0.4987 | 0.2509 | 1.9875 | .0469 | 1.6466 (1.0069−2.6923) |
| Men ≤81.92% (experimental arm) | 0.2285 | 0.1671 | 1.3673 | .1715 | 1.2567 (0.9057-1.7437) |
| Percentage of subsequent PD-(L)1 inhibitor (control arm) | −0.0063 | 0.0083 | −0.7618 | .4462 | 0.9937 (0.9777-1.0101) |
| Percentage of subsequent PD-(L)1 inhibitor (experimental arm) | 0.0362 | 0.0384 | 0.9439 | .3452 | 1.0369 (0.9618-1.1180) |
| **B2. Subsequent PD-(L)1 Inhibitor (*m* = 14)**^††^ | | | | | |
| Intercept | −0.6915 | 0.2426 | −2.8500 | .0044 | 0.5008 (0.3113−0.8058) |
| Add-on (to ChT or MKI) or combination with open-label design | 0.5963 | 0.3476 | 1.7153 | .0863 | 1.8154 (0.9185−3.5880) |
| ECOG PS scores of 0 ≤50.28% (experimental arm) | 0.3748 | 0.2186 | 1.7143 | .0865 | 1.4547 (0.9477−2.2327) |
| Men ≤81.92% (experimental arm) | 0.1871 | 0.1535 | 1.2189 | .2229 | 1.2057 (0.8924−1.6291) |

Abbreviations: ChT, chemotherapy; CI, confidence interval; ECOG PS, Eastern Cooperative Oncology Group performance status; MKI, multikinase inhibitor; PD-(L)1, programmed cell death (ligand)-1

These meta-regression analyses were performed using the escalc() and rma() functions in the metafor package (version 3.8-1) of R 4.2.1.

^†^ The test for residual heterogeneity showed a χ^2^ statistic (df = 8) = 2.56, *P* = .96 and *I*^2^ ≈ 0%. Thus, the linear fixed-effects meta-regression model was reported.

^††^ The test for residual heterogeneity showed a χ^2^ statistic (df = 10) = 3.63, *P* = .96 and *I*^2^ ≈ 0%. Thus, the linear fixed-effects meta-regression model was reported.

**Table 6C.** Sensitivity Analyses for Linear Fixed-Effects Meta-Regression Analysis of Mean Differences in Natural Logarithms of Hazard Ratios for Overall Survival Between Asian and Non-Asian Subgroups

| **Covariates** | **Regression Coefficient Estimate** | **Standard Error** | ***Z* Value** | ***P* Value** | **Estimated Ratio of**  **Hazard Ratios**  **(95% CI)** |
| --- | --- | --- | --- | --- | --- |
| **C1. Any Subsequent Anticancer Therapy and Subsequent PD-(L)1 Inhibitor (*m* = 11)**^¶^ | | | | | |
| Intercept | −0.3602 | 1.0137 | −0.3553 | .7224 | 0.6975 (0.0957-5.0866) |
| Add-on (to ChT or MKI) or combination with open-label design | 0.5577 | 0.4662 | 1.1964 | .2315 | 1.7467 (0.7005-4.3553) |
| ECOG PS scores of 0 ≤50.28% (experimental arm) | 0.4158 | 0.3608 | 1.1525 | .2491 | 1.5156 (0.7473-3.0738) |
| Men ≤81.92% (experimental arm) | 0.1543 | 0.3127 | 0.4935 | .6217 | 1.1668 (0.6322-2.1535) |
| Percentage of any subsequent anticancer therapy (control arm) | −0.0059 | 0.0237 | −0.2486 | .8037 | 0.9941 (0.9489-1.0414) |
| Percentage of any subsequent anticancer therapy (experimental arm) | −0.0005 | 0.0230 | −0.0209 | .9833 | 0.9995 (0.9555-1.0455) |
| Percentage of subsequent PD-(L)1 inhibitor (control arm) | 0.0024 | 0.0122 | 0.1964 | .8443 | 1.0024 (0.9786-1.0268) |
| Percentage of subsequent PD-(L)1 inhibitor (experimental arm) | −0.0012 | 0.0951 | −0.0126 | .9900 | 0.9988 (0.8289-1.2035) |
| **C2. Any Subsequent Anticancer Therapy and Subsequent PD-(L)1 Inhibitor (*m* = 11)**^¶¶^ | | | | | |
| Intercept | −0.6576 | 0.2583 | −2.5457 | .0109 | 0.5181 (0.3123−0.8596) |
| Add-on (to ChT or MKI) or combination with open-label design | 0.5963 | 0.3476 | 1.7153 | .0863 | 1.8154 (0.9185−3.5880) |
| ECOG PS scores of 0 ≤50.28% (experimental arm) | 0.4279 | 0.2225 | 1.9231 | .0545 | 1.5340 (0.9918−2.3729) |
| Men ≤81.92% (experimental arm) | 0.1533 | 0.1773 | 0.8644 | .3874 | 1.1657 (0.8235−1.6499) |

Abbreviations: ChT, chemotherapy; CI, confidence interval; ECOG PS, Eastern Cooperative Oncology Group performance status; MKI, multikinase inhibitor; PD-(L)1, programmed cell death (ligand)-1

These meta-regression analyses were performed using the escalc() and rma() functions in the metafor package (version 3.8-1) of R 4.2.1.

^¶^ The test for residual heterogeneity showed a χ^2^ statistic (df = 3) = 0.43, *P* = .93 and *I*^2^ ≈ 0%. Thus, the linear fixed-effects meta-regression model was reported.

^¶¶^ The test for residual heterogeneity showed a χ^2^ statistic (df = 7) = 0.68, *P* = 1.00 and *I*^2^ ≈ 0%. Thus, the linear fixed-effects meta-regression model was reported.

**Figure 1A.** Random-Effects Meta-analysis of Overall Survival With Knapp-Hartung Adjustment (*m* = 29)


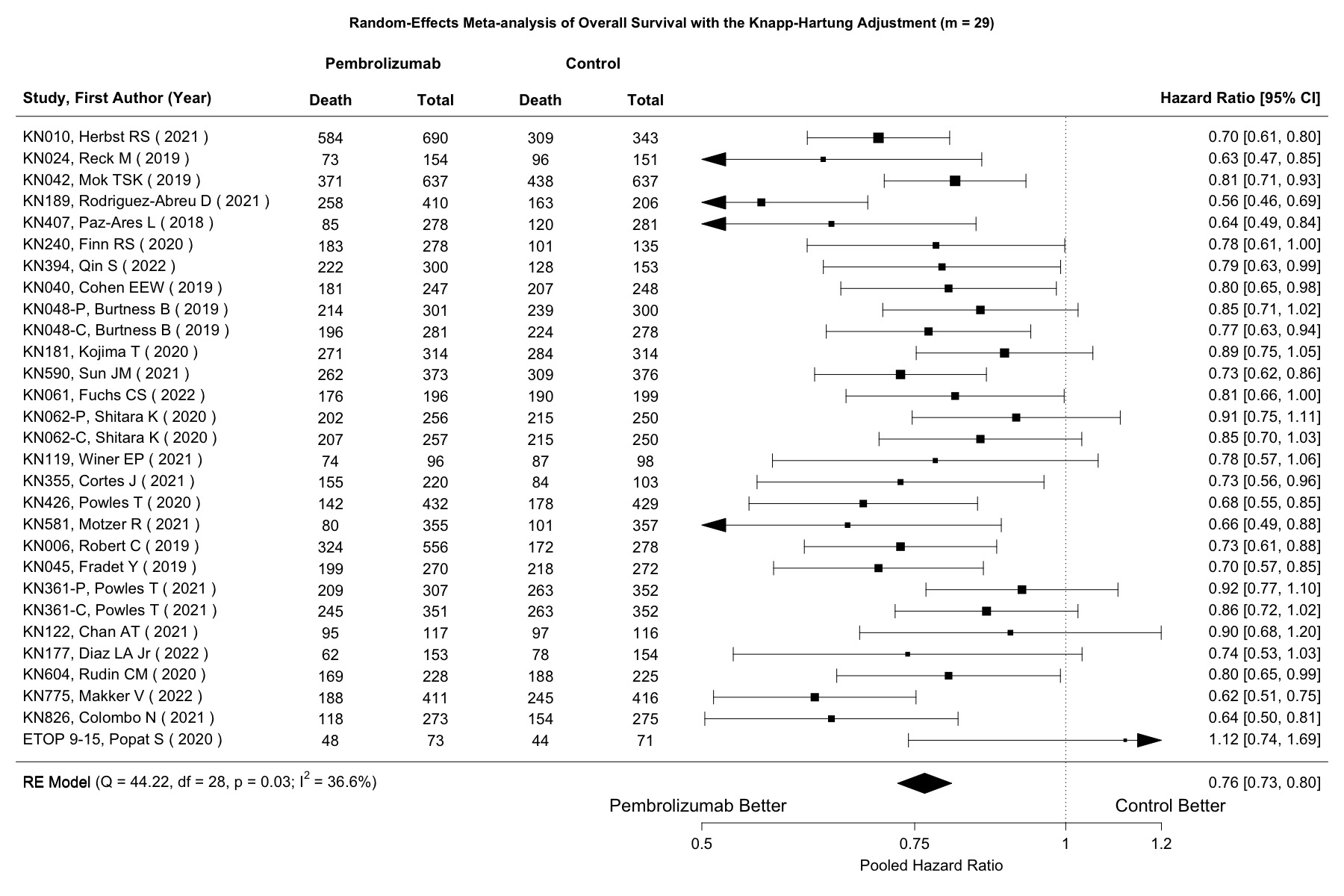


**Figure 1B.** Random-Effects Meta-analysis of Progression-free Survival With Knapp-Hartung Adjustment (*m* = 29)


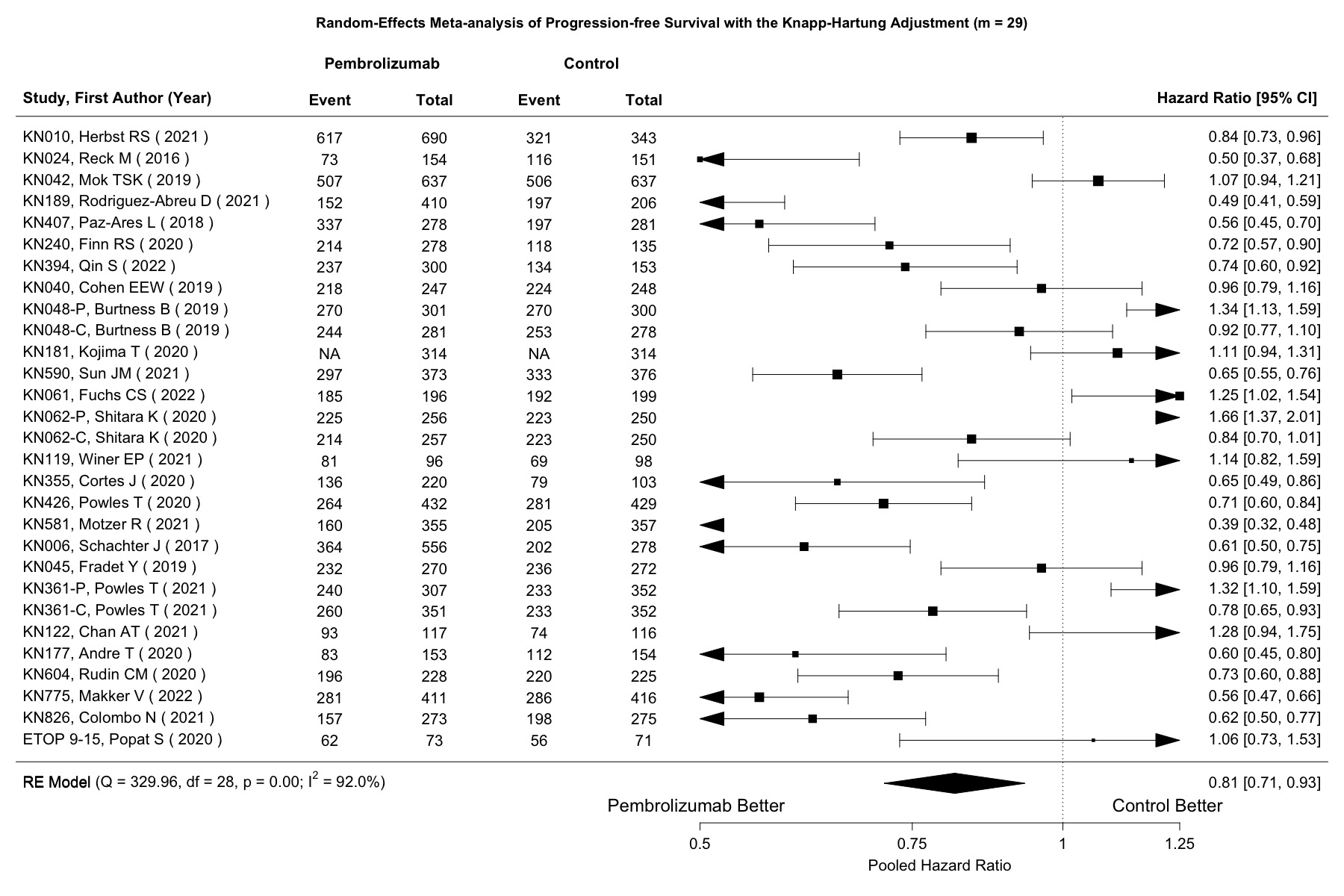


**Figure 2A.** Normal *Q-Q* Plot of the Linear Fixed-Effects Meta-Regression Model for Overall Survival Differences

**
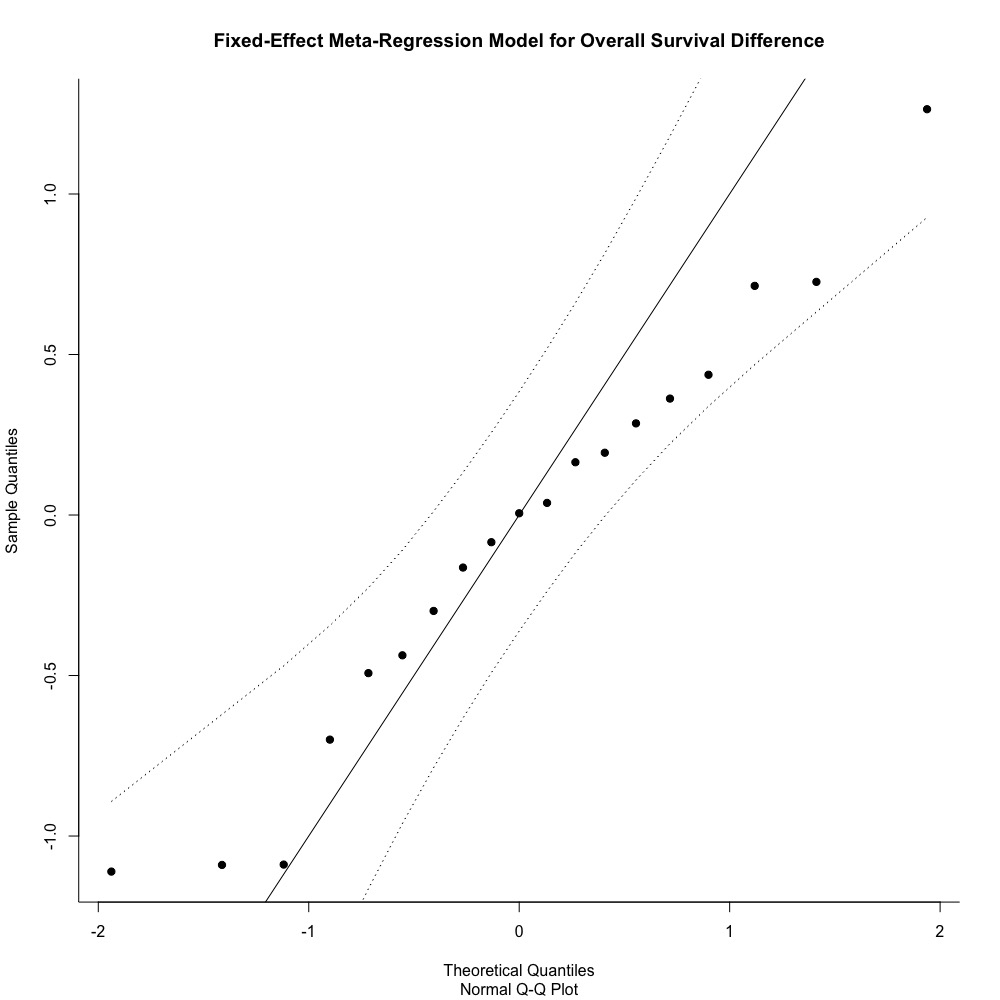
**

**Figure 2B.** Normal *Q-Q* Plot of the Linear Fixed-Effects Meta-Regression Model for Progression-free Survival Differences**
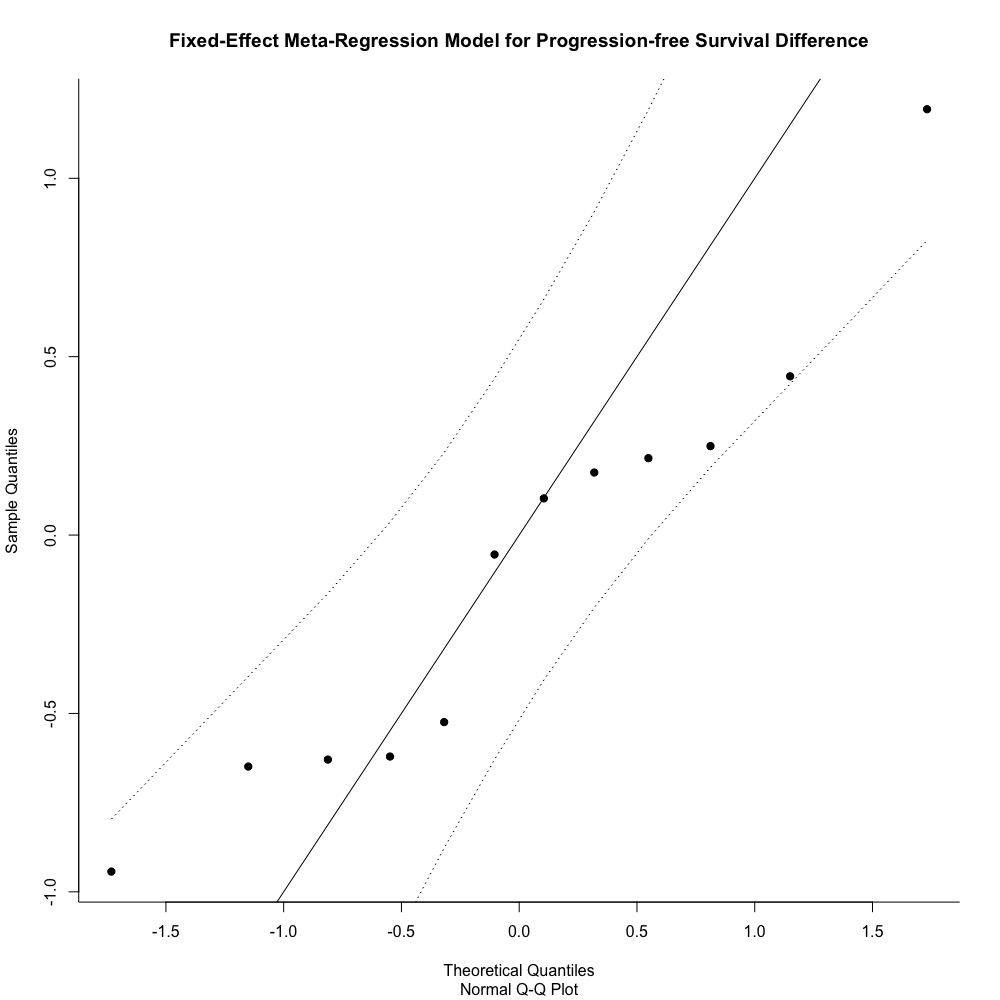
**

**Figure 3A.** Funnel Plot of the Linear Fixed-Effects Meta-Regression Model for Overall Survival Differences

**
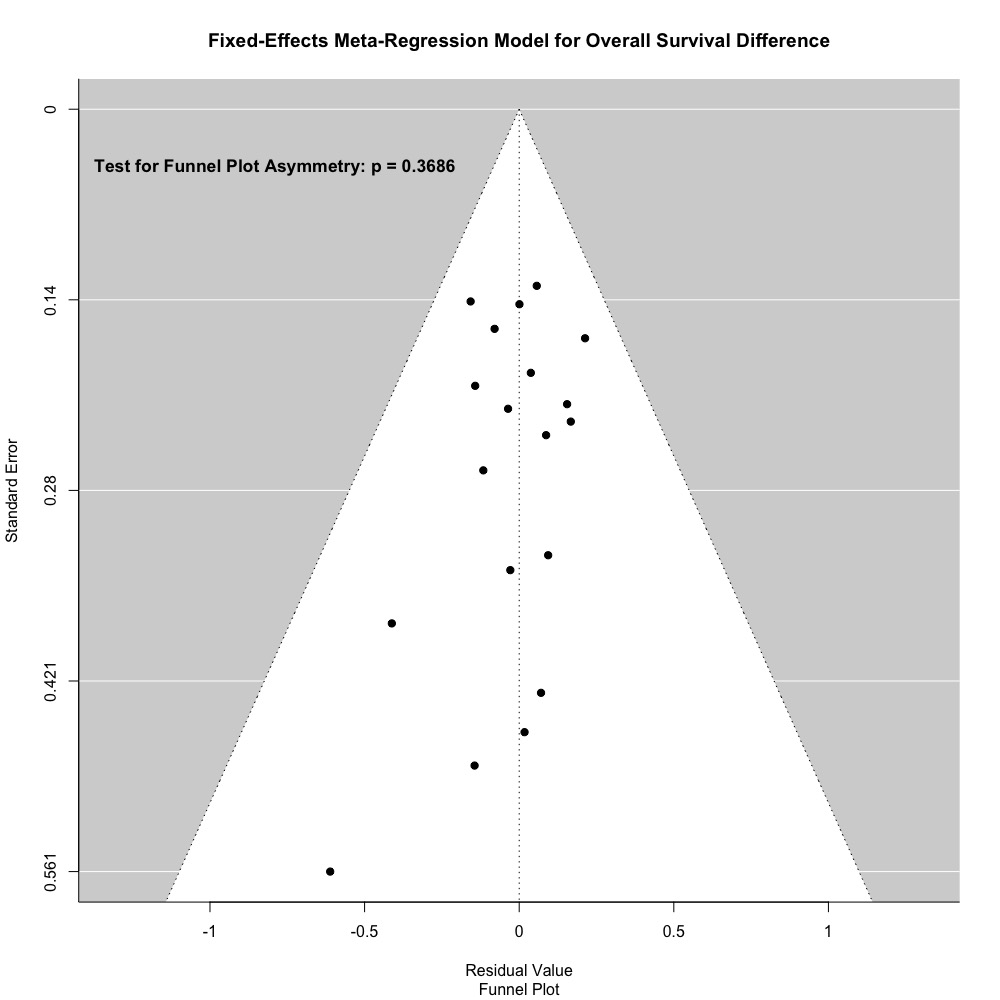
**

**Figure 3B.** Funnel Plot of the Linear Fixed-Effects Meta-Regression Model for Progression-free Survival Differences

**
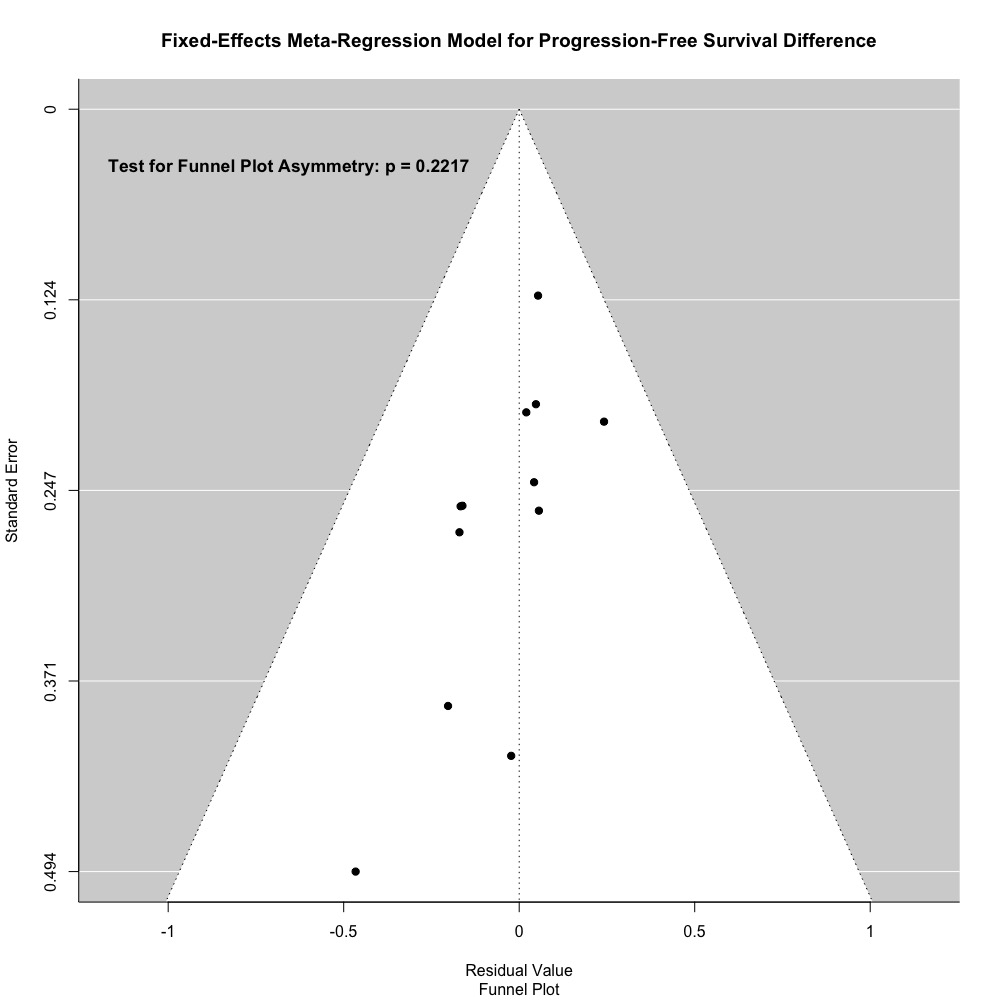
**

**Figure 4A.** Influential Study Diagnostics for the Linear Fixed-Effects Meta-Regression Model for Overall Survival Differences

**
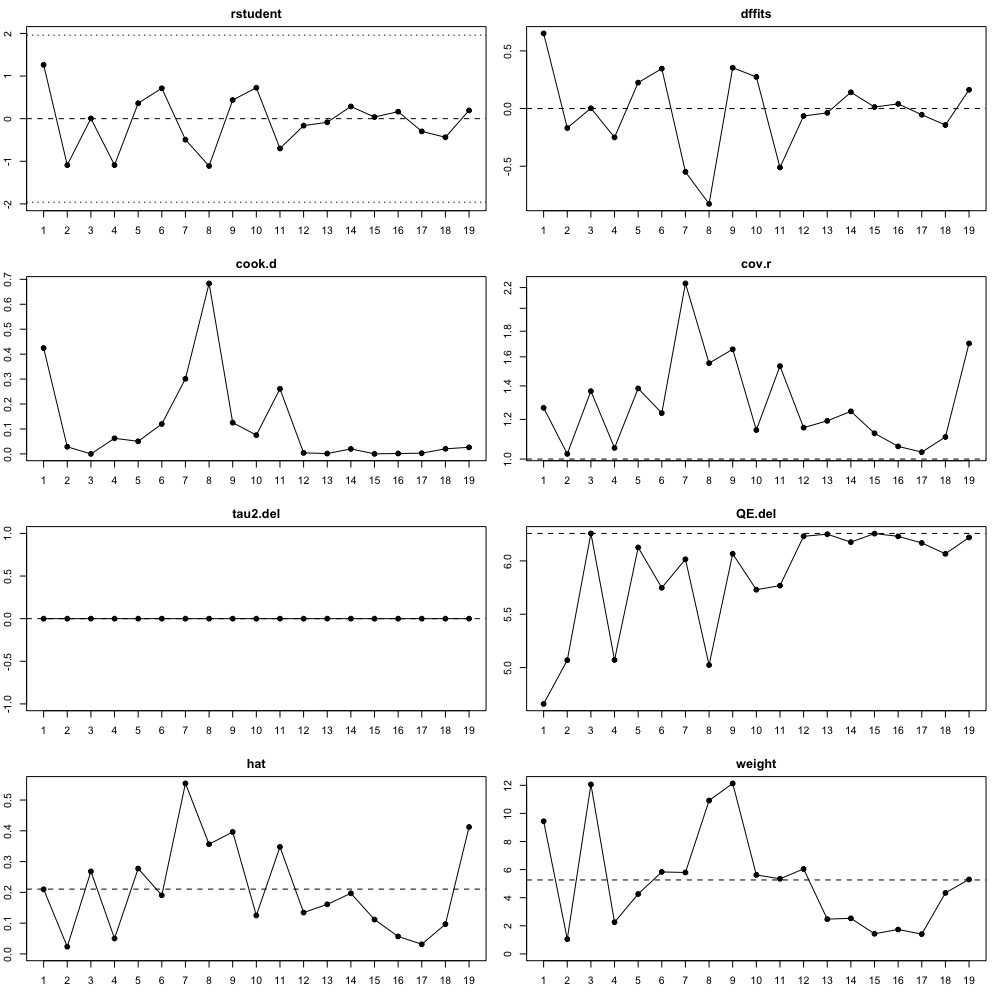
**

**Figure 4A.** Influential Study Diagnostics for the Linear Fixed-Effects Meta-Regression Model for Overall Survival Differences (Continued)

**
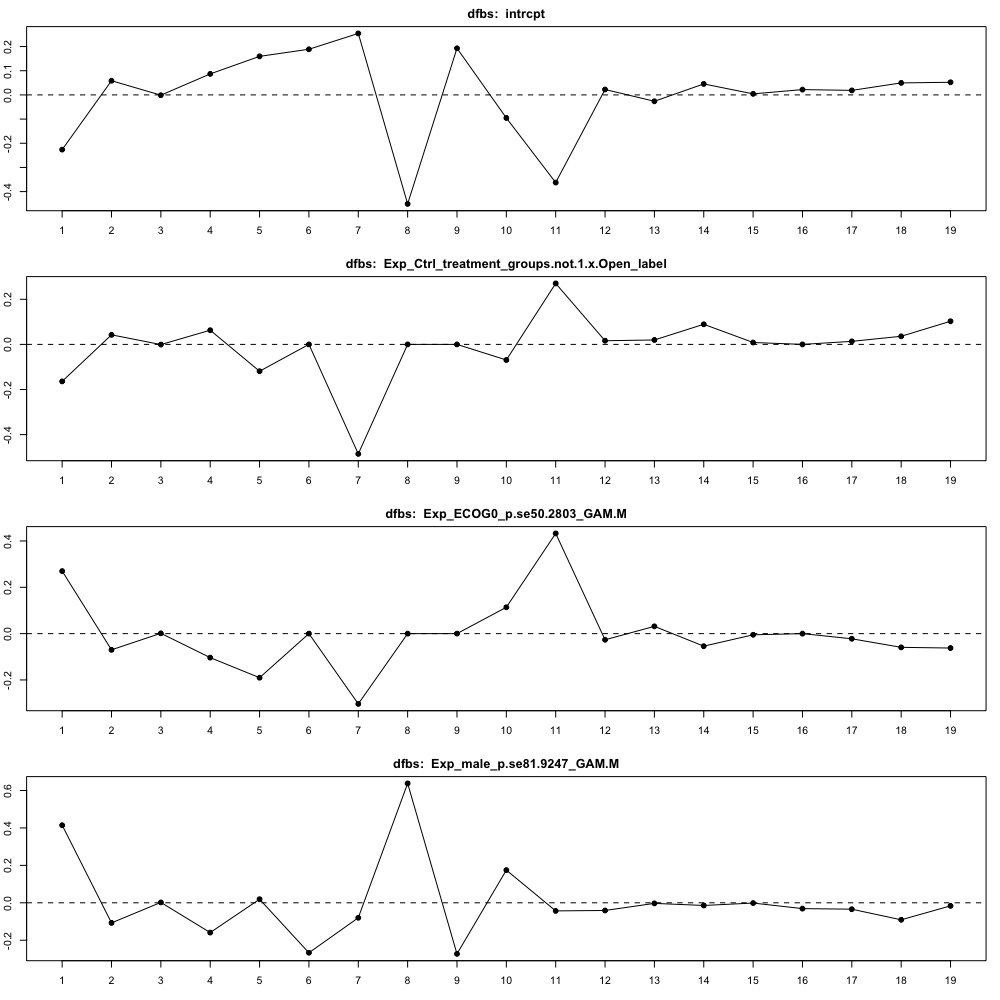
**

**Figure 4B.** Influential Study Diagnostics for the Linear Fixed-Effects Meta-Regression Model for Progression-free Survival Differences

**
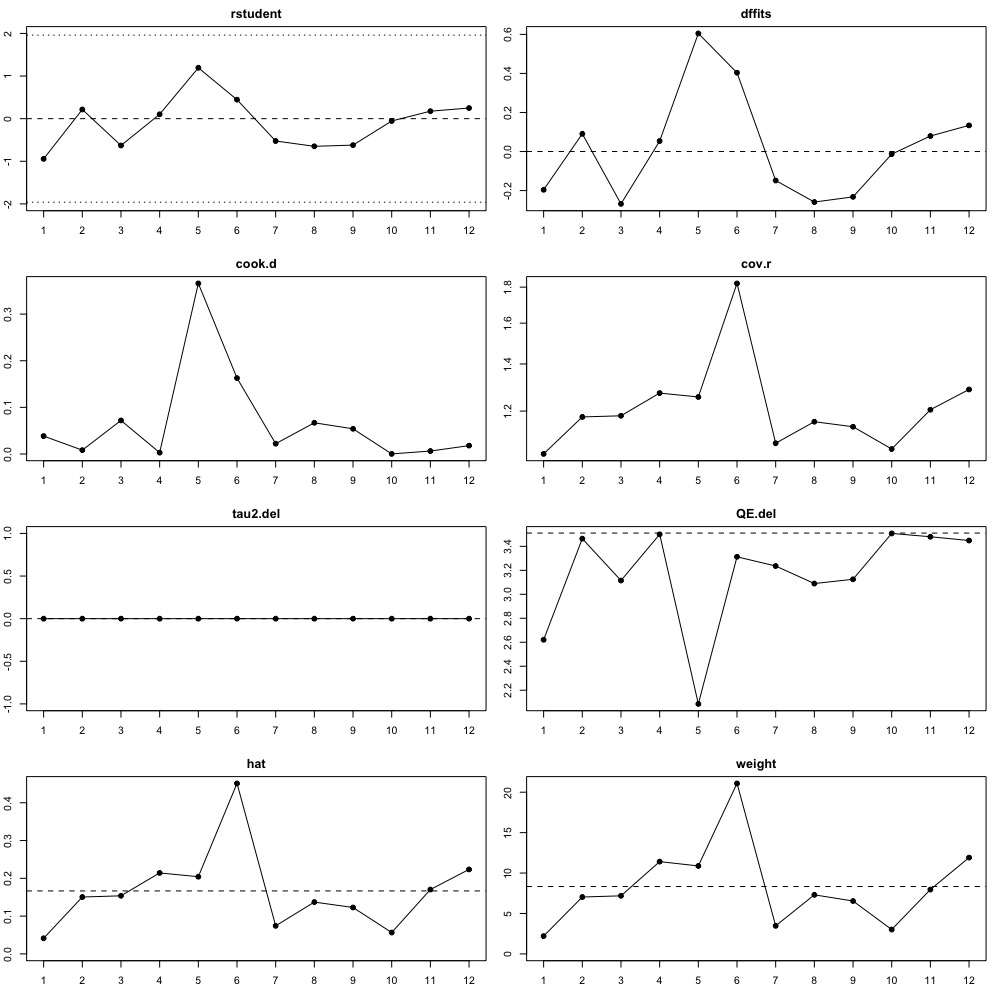
**

**Figure 4B.** Influential Study Diagnostics for the Linear Fixed-Effects Meta-Regression Model for Progression-free Survival Differences (Continued)

**
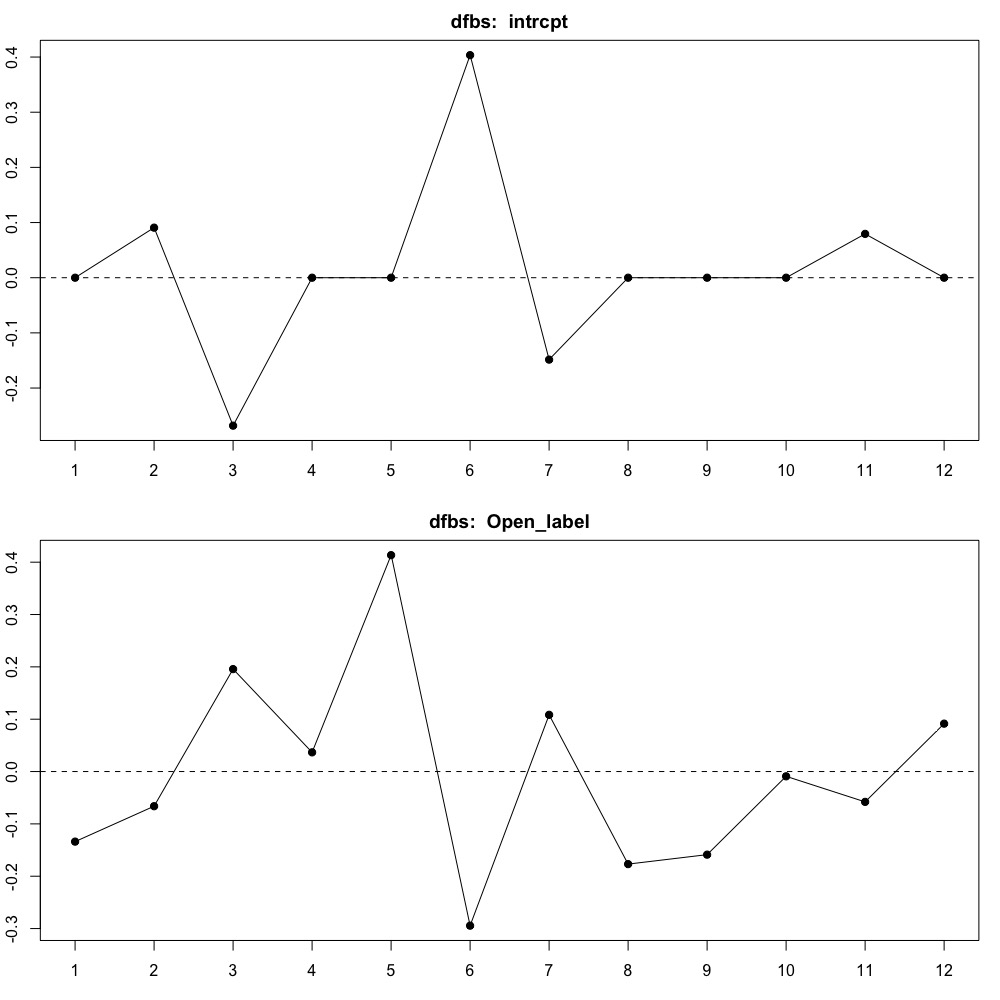
**
